# Administration of turmeric kombucha ameliorates lipopolysaccharide-induced sepsis by attenuating inflammation and modulating gut microbiota

**DOI:** 10.3389/fmicb.2024.1452190

**Published:** 2024-08-30

**Authors:** Jingqian Su, Qingqing Tan, Shun Wu, Fen Zhou, Chen Xu, Heng Zhao, Congfan Lin, Xiaohui Deng, Lian Xie, Xinrui Lin, Hui Ye, Minhe Yang

**Affiliations:** Fujian Key Laboratory of Innate Immune Biology, Biomedical Research Center of South China, College of Life Science, Fujian Normal University, Fuzhou, Fujian, China

**Keywords:** kombucha, turmeric, sepsis, lipopolysaccharide, inflammation

## Abstract

Our research team previously reported the immunomodulatory effects of kombucha fermentation liquid. This study investigated the protective effects of turmeric kombucha (TK) against lipopolysaccharide (LPS)-induced sepsis and its impact on the intestinal microbiota of mice. A turmeric culture medium without kombucha served as the control (TW). Non-targeted metabolomics analysis was employed to analyze the compositional differences between TK and TW. Qualitative analysis identified 590 unique metabolites that distinguished TK from TW. TK improved survival from 40 to 90%, enhanced thermoregulation, and reduced pro-inflammatory factor expression and inflammatory cell infiltration in the lung tissue, suppressing the NF-κB signaling pathway. TK also altered the microbiome, promoting *Allobaculum* growth. Our findings shed light on the protective effects and underlying mechanisms of TK in mitigating LPS-induced sepsis, highlighting TK as a promising anti-inflammatory agent and revealing new functions of kombucha prepared through traditional fermentation methods.

## 1 Introduction

Kombucha, a traditionally steeped fermented tea beverage with an invigorating flavor, is produced from tea and sugar water fermented by a consortium of acetic acid bacteria, yeast, and lactic acid bacteria (Tran et al., 2020). This brew is enriched in tea polyphenols and organic acids, conferring various health benefits, including antibacterial, anti-inflammatory, and immune enhancement effects (Mousavi et al., 2020). Over the past decade, the bioactive attributes of kombucha have increased its popularity, especially in Western countries, as evidenced by a marked increase in its consumption (Cardoso et al., 2021). Turmeric, endowed with bioactive compounds such as polyphenols, curcumin, and its derivatives, is primarily extracted from the rhizome. It is widely used as a culinary spice or natural food colorant (Fadus et al., 2017) and is recognized for its antioxidant, anti-inflammatory, and anticancer activities (Karimian et al., 2017; Yang et al., 2022).

Sepsis, which is characterized by life-threatening organ dysfunction or failure, arises from an imbalance between inflammation and immunosuppression (Huang et al., 2019). The exacerbation of inflammation in sepsis is primarily driven by cytokines, complement components, and the activation of the coagulation system. Lipopolysaccharide (LPS), which is typically injected intraperitoneally, is pivotal in establishing sepsis models (Singer et al., 2016). The LPS-injected mouse model mirrors the clinical manifestations of early-stage sepsis, characterized by the rapid activation of innate and adaptive immune responses. This activation leads to the release of pro-inflammatory factors and lymphocyte apoptosis, culminating in immune dysfunction or suppression (van der Poll et al., 2017).

Recent studies have ventured into alternative fermentable substrates, including diverse teas, medicinal herbs, plant derivatives, and various sugars. Contemporary kombucha variants include fruit juices, leaves, herbal plants, and dairy products. The pharmacological activity of these fermented products is contingent on the composition and concentration of active substances in the fermentation liquid (Su et al., 2023b). For instance, kombucha fermented with substrates, such as oak leaves, has been noted for its anti-inflammatory efficacy (Vázquez-Cabral et al., 2017). Previous studies have suggested that the prolonged oral administration of kombucha can offer protection against LPS-induced sepsis and modulate the gut microbiota (Li L. et al., 2020; Wang et al., 2019; Wang et al., 2021b). Turmeric kombucha (TK) fermentation liquid functioned as an effective immune modulator in animals experimentally infected with *Salmonella typhi* (Zubaidah et al., 2021). However, the potential for using TK as a novel approach for sepsis prevention and treatment remains unclear.

Preliminary findings from our research group have revealed that TK has notable anti-inflammatory effects at the cellular level (Su et al., 2023e). Here, to further elucidate its potential for long-term prevention of and protection against sepsis, we utilized an LPS-induced sepsis mouse model to investigate the anti-inflammatory mechanisms of TK.

By connecting bioactive metabolites, gut microbiota modulation, and anti-inflammatory effects, our study offers deeper insights into the mechanisms underlying TK against sepsis. This study not only expands the research on kombucha and turmeric but also suggests that TK could be a promising candidate for long-term prevention and treatment of sepsis, with potential applications in the development of functional beverages.

## 2 Materials and methods

### 2.1 Chemicals and reagents

Unless otherwise specified, all chemicals were sourced from Sigma (St. Louis, MO, USA). Turmeric powder was obtained from Guangdong Fengchun Pharmaceutical Co. Ltd (Zhanjiang, China). White sugar was sourced from Shanghai Tangjiu Co., Ltd (Shanghai, China). Lapsang Souchong black tea (*Camellia sinensis*), characterized by its curled shape, was provided by Fujian Agricultural Reclamation Tea Industry Co., Ltd. The reverse transcription kit, Green qPCR SuperMix, and the Bicinchoninic Acid Protein Concentration Kit were acquired from TransGen Biotech Co., Ltd (Beijing, China). ELISA kits for TNF-α, IL-1β, and IL-6 were procured from Shanghai Excell Biological Technology Co., Ltd (Shanghai, China). All other reagents were of domestic analytical grade.

### 2.2 Starter culture

The kombucha starter strain, obtained from a local village in Zhangzhou, Fujian Province, China, was cultured and maintained at 25°C, according to the methodology outlined by Wang et al. (2021a).

The starter culture was prepared by inoculating the kombucha strain into a medium with the following composition: 15.0 g/L turmeric powder, 5.0 g/L black tea, and 35.0 g/L white sugar. This mixture was dissolved in boiling water to a final volume of 1 L and allowed to cool before inoculation. The culture was then incubated for 7 days and re-cultured weekly until stable growth of the kombucha starter culture was achieved.

### 2.3 TK preparation

TK was prepared by dissolving 35 g/L white sugar and 20 g/L turmeric in water, followed by inoculation with a 15% kombucha starter culture. The samples were fermented at 28°C for 7 days and sealed with eight layers of sterilized gauze. Turmeric culture medium without added kombucha served as the control (TW).

### 2.4 Sample preprocessing and analysis

Samples were mixed thoroughly in a sterile environment, filtered using a 0.22-μm microporous membrane to eliminate microbes and insoluble impurities, and stored at −80°C in sterile tubes.

### 2.5 Non-targeted metabolomics and data processing

The samples were subjected to liquid chromatography–mass spectrometry analysis at Shanghai Sangon Biotech Co., Ltd. The non-targeted metabolomics analysis was performed using an ACQUITY UPLC I-Class plus system coupled with a high-resolution QE mass spectrometer. Chromatographic separation was achieved using an ACQUITY UPLC HSST3 column (100 mm × 2.1 mm, 1.8 μm) at 45°C, with a mobile phase consisting of water with 0.1% formic acid (Phase A) and acetonitrile with 0.1% formic acid (Phase B), at a flow rate of 0.35 mL/min and an injection volume of 2 μL. Mass spectrometric data were acquired using an electrospray ionization (ESI) source in both positive and negative ion modes. Data were collected using UNIFI 1.8.1 software and analyzed using Progenesis QI v2.3 software, covering baseline correction, peak identification, and normalization. Compounds were identified using the Human Metabolome Database (HMDB), Metlin, and Lipid Map (v2.3) databases. Principal component analysis (PCA) was used to assess overall sample distribution and stability, whereas orthogonal partial least squares discriminant analysis (OPLS-DA) and partial least squares discriminant analysis (PLS-DA) were used to identify metabolic profile differences and differential metabolites. The Pearson correlation coefficient was used to analyze metabolite correlations, and the Kyoto Encyclopedia of Genes and Genomes (KEGG) database facilitated metabolic pathway enrichment analysis.

### 2.6 Animals

Specific pathogen-free C57BL/6 adult mice (age, 8–10 wk; *n* = 160; balanced sex ratio), each weighing 20 ± 1 g, were obtained from the Animal Facility of Fujian Normal University, China. The environmental conditions were 23–25°C, 40–60% humidity, and the mice had access to sufficient water and food under a 12-h light/dark cycle.

### 2.7 *In vivo* experiments

Following a 7-day acclimatization period, the mice were randomly allocated to four groups (*n* = 20 each, balanced sex ratio): sham, oral TK, LPS-induced sepsis (LPS), and TK + LPS. Drug efficacy studies of sepsis typically require a mortality rate in animal models of at least 50% (Russell, 2006).

The sham and LPS groups received 1.0 μL/g/d oral saline daily. The TK and TK + LPS groups were similarly administered TK fermentation liquid (1.0 μL/g/d). After one month of daily saline or TK administration, the LPS and TK + LPS groups received an intraperitoneal injection of 15 mg/kg LPS to induce sepsis, whereas the sham and TK groups received a similar volume of saline. Samples were collected 12 h after the induction of sepsis for further analysis.

Sepsis severity was evaluated using the murine sepsis score (MSS; Shrum et al., 2014). Rectal temperatures were monitored every 4 h over a 48-h period using the TH212 intelligent digital thermometer from Beijing Zhongjiao Building Instrument Technology Development Co., Ltd (Beijing, China). For anesthesia, the mice received an intraperitoneal injection of pentobarbital sodium salt. Subsequently, blood, fecal, ascitic fluid, and various tissue samples were collected according to previously established protocols (Wang et al., 2021a).

### 2.8 Western blotting

Western blotting was performed using established protocols (Su et al., 2023a), including protein extraction, electrophoresis, membrane transfer, and antibody incubation. After washing, secondary antibody incubation and chemiluminescence visualization were performed. Protein band intensities were quantified and expressed as fold change relative to the control samples. The antibodies used are listed in Table 1.

**TABLE 1:** Antibodies used in the study.

Antibody	Manufacturer	Identifier
NF-κB	ABclonal	A19653
p-NF-κB	ABclonal	AP0475
IκBα	Abmart	Cat#T55026
p-IκBα	ABclonal Technology	Cat#AP0999
GAPDH	ABclonal	A19056
CD45 BUV395-A	Becton, Dickinson and Company	Cat#564279
CD45R BB515-A	Becton, Dickinson and Company	Cat#553088
CD3 APC-Alexa 700-A	Becton, Dickinson and Company	Cat#561388
CD8 BUV805-A	Becton, Dickinson and Company	Cat#612898
CD25 BB700-A	Becton, Dickinson and Company	Cat#567482
NK1-1 PE-CF594-A	Becton, Dickinson and Company	Cat#562864
CD69 BV711-A	Becton, Dickinson and Company	Cat#740664
CD4 BUV496-A	Becton, Dickinson and Company	Cat#612952
CD120b BV421-A	Becton, Dickinson and Company	Cat#564088
CD127 PE-Cy7-A	Becton, Dickinson and Company	Cat#560733
CD20 PE-BYG568-A	BioLegend	Cat#152106

### 2.9 RT-qPCR

Total RNA extraction was performed utilizing the TRIzol reagent from Takara (Tokyo, Japan). RT-qPCR was conducted using specific primers (Table 2), and levels were normalized against those of the housekeeping gene, *Gapdh*, as previously described (Su et al., 2023a). The mRNA levels were quantified as the fold change relative to the control using the ΔΔCt method. PCR was replicated three times for each gene, and the mean Ct value was used to assess gene expression stability.

**TABLE 2:** Sequences of primers used for *q*RT-PCR.

Primer	Sequence (5′–3′)
IL-1β	F: TCATTGTGGCTGTGGAGAAG
IL-1β	R: TCATCTCGGAGCCTGTAGTG
TNF-α	F: GCCTCCCTCTCATCAGTTCTA
TNF-α	R: GGCAGCCTTGTCCCTTGA
IL-6	F: CTTGGGACTGATGCTGGTG
IL-6	R: TCATTTCCACGATTTCCCA
GAPDH	F: AGAGTGTTTCCTCGTCCCG
GAPDH	R: GATGGCAACAATCTCCACTTT

GAPDH, glyceraldehyde 3-phosphate dehydrogenase; IL, interleukin; TNF, tumor necrosis factor; F, forward; R, reverse.

### 2.10 ELISA

Cytokine concentrations (IL-1β, IL-6, and TNF-α) were quantified employing ELISA kits (IL-1β: SMLB00C, IL-6: SM6000B, TNF-α: SMTA00B, R&D Systems, MN, USA) in accordance with the manufacturer’s instructions.

### 2.11 Histopathological analysis

Histopathological examination was performed using standard hematoxylin and eosin (H&E) staining protocols (Qu et al., 2022). Lung tissue was washed with sterile saline and fixed in 4% paraformaldehyde for 24 h. The samples underwent dehydration, were sectioned into 4-μm slices, and were mounted on slides. The slides were deparaffinized at 60°C, rehydrated, and stained with H&E. After staining, the sections were rinsed with ethanol and xylene before cover slipping. Tissue injury and necrosis levels were evaluated using a 0–4 grading system (Su et al., 2023a).

### 2.12 Flow cytometry

Flow cytometry was conducted according to established methods (Su et al., 2023a), using the antibodies listed in Table 1. Red blood cells were lysed, and the samples were resuspended in 2% PBS buffer (HyClone, UT, USA) for cell counting. Samples were surface-stained with specific antibodies (Table 1) in staining buffer at 4°C for 30 min. Analysis was performed using FACSymphony A5 (BD Biosciences, San Diego, CA, USA), and data were analyzed using FlowJo software v10.5.3 (FlowJo LLC, Ashland, OR, USA).

### 2.13 16S rRNA sequencing and bioinformatics

Twelve hours after LPS administration, the mice were euthanized via cervical dislocation. After disinfecting the exterior with 75% alcohol, the abdominal cavities were opened to collect fecal samples from the cecum to the rectum, which were stored in sterile Eppendorf tubes (to ensure robust results, five replicates were used per group). Sample analysis was carried out by Beijing Biomarker Technologies Co., Ltd. (Beijing, China), employing established protocols for DNA extraction and *16S* rRNA sequencing (Li Y. et al., 2020) using a Magnetic Soil and Stool DNA kit (Cat. 4992738, Tiangen Biotech Co., Ltd., Beijing, China) and universal primers 27F and 1492R with PacBio barcodes. The PacBio Sequel II platform was utilized for library construction, sequencing, and subsequent bioinformatics analysis on the BMK Cloud platform.

The linear discriminant analysis (LDA) effect size (LEfSe) method was employed to quantify the notable differences in taxonomy by calculating the effect size using LDA and setting the discriminative feature’s logarithmic LDA score threshold at 4.0. Redundancy analysis was conducted within the R environment, utilizing the vegan package (version 2.3), to scrutinize the microbial diversity across various factors. Furthermore, to ascertain the impact of kombucha on gut metabolism through differences in KEGG pathways among groups, a phylogenetic investigation of communities by reconstruction of unobserved states (PICRUSt) analysis was performed utilizing STAMP (version 2.1.3),^1^ leveraging *16S* rDNA datasets.

### 2.14 Whole-genome sequencing

RNA sequencing was performed at Beijing BioMarker Bioinformatics Technology Co., Ltd. using an Illumina NovaSeq 6000 platform, according to established comprehensive genome sequencing protocols (Zhang et al., 2021).

### 2.15 Statistical analyses

Image processing was performed using Photoshop, Illustrator 2020 (Adobe Illustrator), and ImageJ v1.8.0 (NIH, MD, USA). Statistical analyses were executed using GraphPad Prism (v8.0), employing methods such as unpaired two-tailed *t*-tests, one-way ANOVA, two-way ANOVA, or Mantel–Cox tests. The results are presented as the mean ± standard deviation, with significance set at *P* < 0.05.

## 3 Results

### 3.1 Multivariate metabolite and metabolic pathway analysis

**FIGURE 1:**
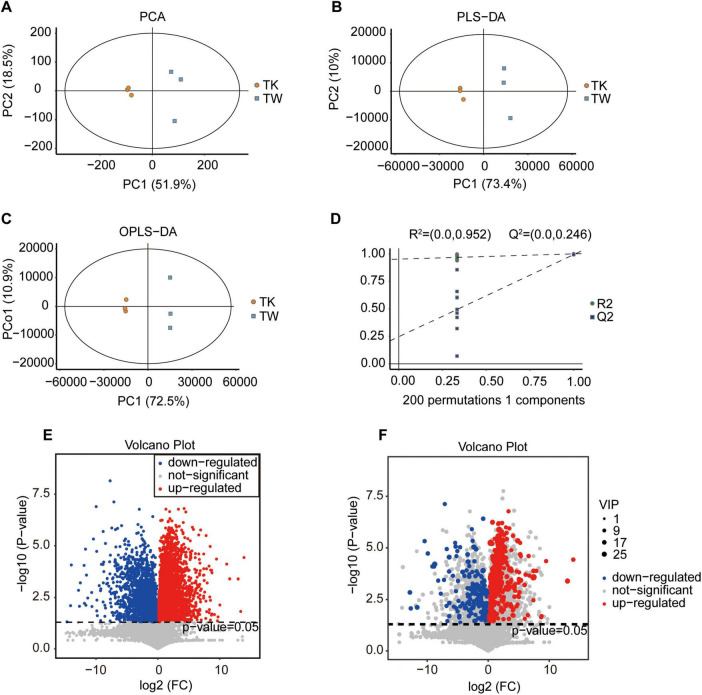
Comparative analysis of substance composition in the turmeric alone (control, TW) and turmeric kombucha (TK), via non-targeted metabolomics (*n* = 3). **(A–D)** Multivariate statistical analysis of TW and TK. **(A)** Principal component analysis (PCA). **(B)** Partial least squares discriminant analysis (PLS-DA). **(C)** Orthogonal partial least squares discriminant analysis (OPLS-DA). **(D)** Permutation test. **(E)** Volcano plot of the differential metabolites in TW and TK. **(F)** Volcano plot depicting variation in metabolites between TW and TK. VIP, variable independent parameter.

Non-targeted metabolomics was used to analyze the metabolic substances and pathways of turmeric with and without fermentation in kombucha (TK and TW, respectively). Advanced statistical techniques, such as PCA, PLS-DA, and OPLS-DA, were used for grouping samples and analyzing metabolic changes (Figures 1A-C). Considerable differences in metabolic composition were observed between TK and TW. The response permutation test of the OPLS-DA model confirmed its accuracy and the absence of overfitting (Figure 1D). *T*-tests and fold-change analysis were used to identify metabolites that were significantly upregulated, downregulated, or unchanged (Figures 1E, F).

**FIGURE 2:**
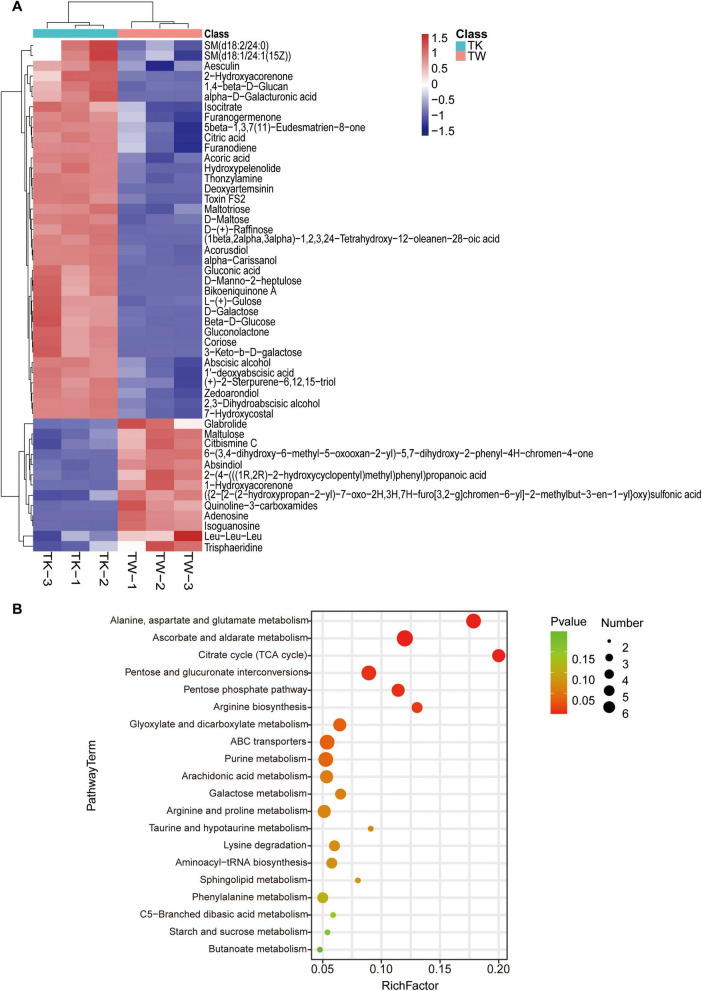
The differential metabolites and pathway enrichment analysis between the turmeric alone (control, TW) and turmeric kombucha (TK). **(A)** Bidirectional clustering analysis of the 50 top differential metabolites in TW and TK. **(B)** Bubble plot enrichment analysis of the 20 top metabolic pathways in TW and TK.

By combining multidimensional and unidimensional analyses, 590 differential metabolites were identified, encompassing 12 categories (Supplementary Table 1). Hierarchical clustering and variable interdependent parameter analysis, used to visually represent these metabolites (Figure 2A), revealed significant post-fermentation changes, notably in 1,4-beta-D-glucan (VIP, 28.14). Categorization of the differential metabolites revealed various differentially expressed compounds, including carbohydrates and their analogs, glycosides, organic acids, polyphenols, flavonoids, alkaloids, and sesquiterpenes (Supplementary Tables 2-7). A summary of 25 turmeric-related compounds was compiled (Supplementary Table 8), and 12 showed significant variance. Pearson’s correlation coefficient analysis was used to explore the linear relationships between these metabolites (Supplementary Figure 1). KEGG database analysis provided insights into metabolic pathway changes during fermentation (Figure 2B and Supplementary Table 9), highlighting significant differences in specific metabolic pathways.

### 3.2 Efficacy of TK in LPS-induced sepsis in mice

**FIGURE 3:**
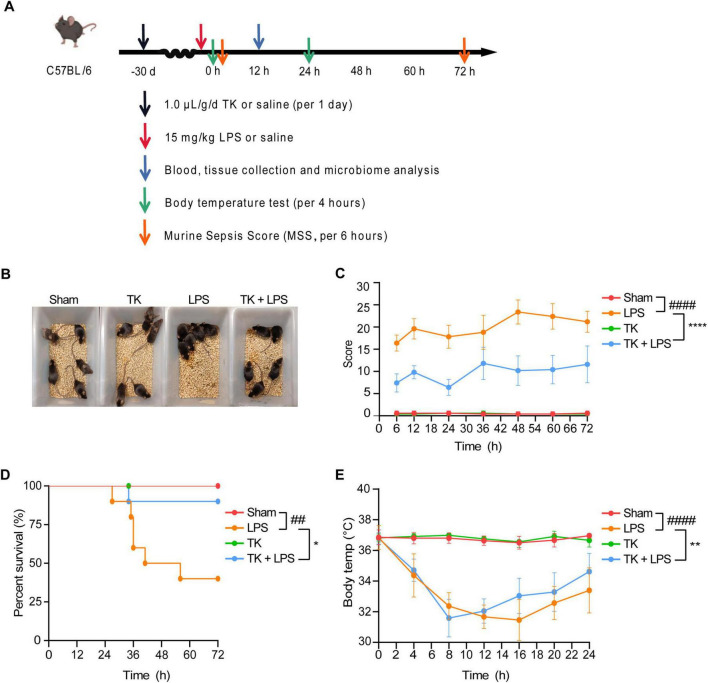
Efficacy of turmeric kombucha (TK) in ameliorating LPS-induced sepsis in mice. **(A)** Timeline of the experimental protocol for development of the LPS-induced sepsis model in mice. **(B)** Observable behavioral alterations in mice 12 h after LPS administration (15 mg/kg). **(C)** Assessment of murine sepsis scores (MSS) in affected mice (*n* = 10). **(D)** Effect of pre-administration of TK on survival rates in septic mice (*n* = 20). **(E)** Effect of TK pre-administration on body temperature regulation in sepsis-affected mice. Statistical analysis: Mantel–Cox test for **(B)**, two-way ANOVA with Bonferroni post-hoc test for **(C–E)**. Significance levels: ^##^*P* < 0.01 and ^####^*P* < 0.0001 against the sham group. *P < 0.05, **P < 0.01, and ****P < 0.0001 against the LPS group. Sample counts are provided within parentheses.

A mouse model of LPS-induced sepsis was used to evaluate the therapeutic efficacy of TK (Figure 3A). Among the LPS-induced septic mice, the TK-treated group exhibited less agglomeration and greater physical status, activity, and appetite than the untreated LPS group (Figure 3B). TK treatment notably reduced LPS-induced MSS (*P* < 0.0001, Figure 3C). Among the septic mice, the TK-treated group exhibited better survival than the LPS group (90 vs. 40%, *P* < 0.05; Figure 3D). Eight hours after sepsis onset, the body temperature of the TK-treated mice normalized swiftly (*P* < 0.01, Figure 3E). This suggests that TK has a significant preventative effect against LPS-induced sepsis in mice.

### 3.3 TK-mediated mitigation of LPS-induced pathology

**FIGURE 4:**
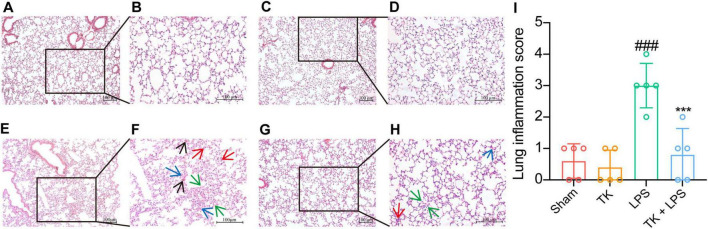
Turmeric kombucha exhibits protective effects against lung tissue damage in LPS-induced septic mice at 12 h. **(A–H)** Representative microscopic images of the lung tissue of mice with LPS-induced sepsis after hematoxylin and eosin (H&E) staining (magnification, 200×). H&E staining reveals the presence of inflammation (indicated by the red arrow), edema (indicated by the blue arrow), hemorrhage (indicated by the black arrow), and thickening of alveolar septa (indicated by the green arrow) in the lung tissues. **(I)** Lung inflammation scores of the lung tissue of LPS-induced septic mice. Results are expressed as the mean ± standard deviation. Data interpretation was conducted using ANOVA followed by Tukey’s post-hoc test (*n* = 5). ^###^*P* < 0.001 vs. the sham group. ***P < 0.001 vs. the LPS group. The experiment was independently replicated five times (scale bars = 100 μm).

To assess the prophylactic effect of TK on LPS-induced sepsis, lung tissue was subjected to H&E staining 12 h post-LPS induction. Figure 4 reveals that TK treatment mitigated lung injury, including reducing alveolar wall thickening and LPS-induced hemorrhage (*P* < 0.001). These results reveal a marked reduction in LPS-induced pulmonary impairment in mice, attributable to the prophylactic properties of TK.

### 3.4 TK significantly reduces inflammatory cytokine levels in LPS-induced septic mice

**FIGURE 5:**
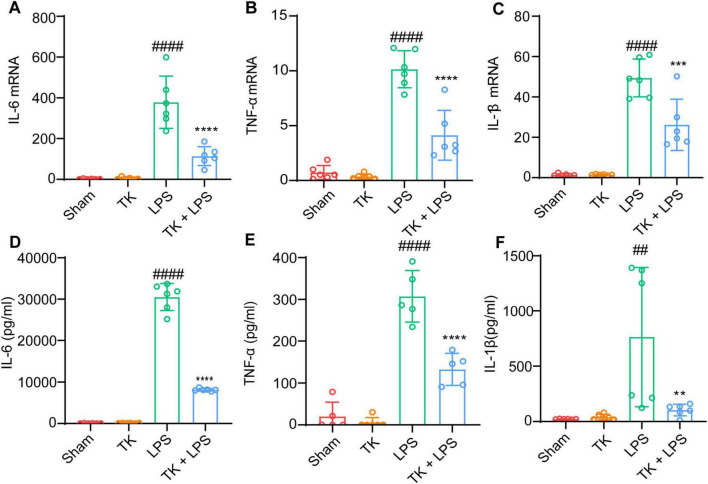
Efficacy of turmeric kombucha in reducing inflammatory factor levels in the serum of LPS-induced septic mice. **(A–C)** IL-6, IL-1β, and TNF-α mRNA levels in mouse lung tissue, examined via RT-qPCR 12 h after LPS administration (15 mg/kg). **(A)** IL-6, **(B)** IL-1β, and **(C)** TNF-α. **(D–F)** IL-6, IL-1β, and TNF-α protein concentrations in the lung tissue determined using ELISA, 12 h after LPS exposure. ANOVA, complemented by Tukey’s *post hoc* test, was employed for data analysis (*n* = 5). ^##^*P* < 0.01 and ^####^*P* < 0.0001 against the sham group. **P < 0.01, ***P < 0.001, and *⁣*⁣**P < 0.0001 against the LPS group.

To elucidate the role of TK in attenuating inflammatory cytokine expression *in vivo*, we evaluated its effect on serum and lung-tissue cytokine levels 12 h post-LPS induction. Using RT-qPCR, we investigated the inhibitory influence of TK on the mRNA expression of inflammatory cytokines (IL-1β, IL-6, and TNF-α) in the lungs post-LPS induction. Inflammatory cytokine expression was significantly lower in the septic mice that received TK than in those that did not (*P* < 0.001, Figure 5A-C). ELISA testing demonstrated a significant reduction in LPS-induced IL-1β, IL-6, and TNF-α serum levels 12 h after LPS-induction (*P* < 0.01, Figures 5D–F). These findings suggest the notable inhibition of inflammatory cytokine upregulation in LPS-induced sepsis model mice, mediated by TK treatment.

### 3.5 Effect of TK on immune cell dynamics in LPS-induced sepsis in mice

**FIGURE 6:**
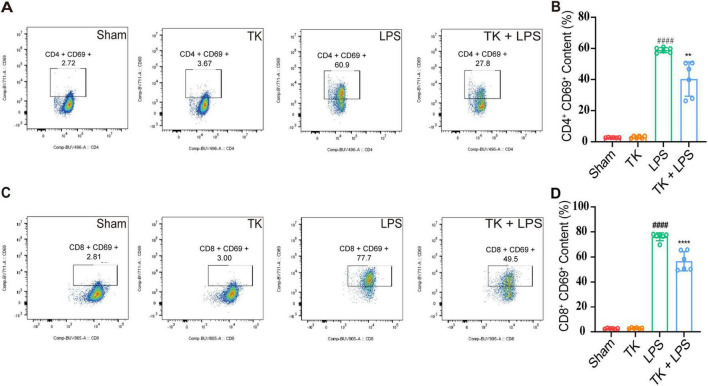
Effect of the turmeric kombucha treatment on the levels of activated CD4+ and CD8+ T cells in the peripheral blood of septic mice. **(A)** Flow cytometry and specific markers used for evaluating activated CD4+ T cells in mice with sepsis. **(B)** Number of activated CD4+ T cells. **(C)** Flow cytometry and markers for assessing activated CD8+ T cells in the same cohort. **(D)** Number of activated CD8+ T cells. Data were analyzed using ANOVA and Tukey’s *post hoc* test (*n* = 5). ^####^*P* < 0.0001 against the sham group. **P < 0.01 and *⁣*⁣**P < 0.0001 against the LPS group.

For the LPS-induced septic mice, the effect of TK on peripheral blood circulating immune cell levels was quantified via flow cytometry. We examined whether TK treatment enhances the immune response in the sepsis model. Twelve hours after LPS induction, the TK group exhibited notably lower populations of activated CD4+ T cells (*P* < 0.01, Figures 6A, B) and CD8+ T cells (*P* < 0.001, Figures 6C, D) than the non-TK group. However, 12 h after LPS induction, the levels of B220, B, CD4+ T, CD8+ T, activated CD4+ T, activated CD8+ T, NK, activated NK, Treg, and activated Treg cells did not differ significantly (*p* > 0.05, Supplementary Figure 2) between the TK and non-TK groups. These findings suggest that TK selectively affects activated CD4+ T and CD8+ T cell populations in LPS-induced sepsis in mice, highlighting its potential modulatory role in specific immune responses.

### 3.6 TK-mediated inhibition of LPS-induced inflammation

**FIGURE 7:**
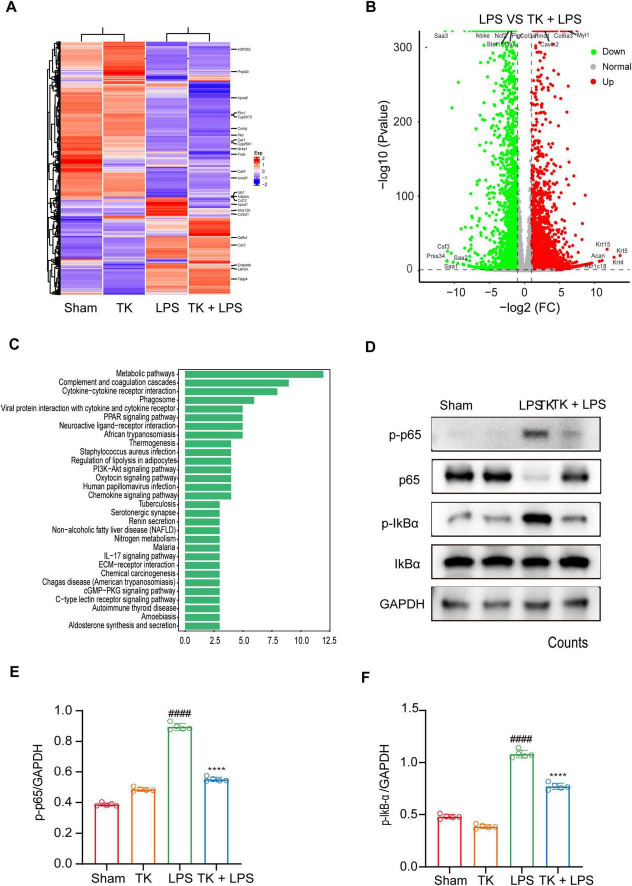
Effect of turmeric kombucha (TK) on the NF-κB pathway in LPS-induced septic mice. **(A–C)** Gene profile analysis in non-septic mice (sham and TK) and LPS-induced septic mice (with or without TK). **(A)** Clustered heatmap of gene expression in the sham, TK, LPS, and LPS + TK groups. **(B)** Volcano plot of differentially expressed genes (DEGs) between LPS-induced septic mice treated with or without TK (LPS vs. LPS + TK; downregulated, blue; upregulated, red; insignificantly altered, black). **(C)** KEGG pathway analysis of DEGs (LPS vs. LPS + TK). **(D–F)** Effect of TK on NF-κB signaling in LPS-induced septic mice. **(D)** Western blot analysis was conducted for NF-κB inhibitor α (IκBα), p-IκBα, NF-κB (p65), and p-p65 in lung tissues 12 h post LPS administration (15 mg/kg). **(E,F)** Densitometric quantification of proteins using ImageJ. One-way ANOVA, followed by Tukey’s *post hoc* test, was applied (*n* = 5). ^####^*P* < 0.0001 against the sham group. *⁣*⁣**P < 0.0001 against the LPS group.

Comprehensive whole-genome RNA sequencing was used to elucidate the mechanistic role of TK in the defense against sepsis. The heatmaps (Figures 7A, B) reveal alterations in gene expression, indicating key differentially expressed genes (DEGs). TK significantly downregulated key genes in various pathways: NF-κB (ikbkb), activation of T cells (lat), and caspase (card14). Volcano plot analysis integration revealed protein interactions (Figure 7C). Subsequent GO and KEGG analyses of the DEGs revealed the regulatory impact of TK. LPS markedly intensified cytokine interactions and pathways. Conversely, TK substantially reduced inflammation (Figures 7C, D). TK notably attenuated the LPS-induced phosphorylation of NF-κB (p65) and IKBα, indicating reduced NF-κB activation (*P* < 0.001, Figures 7E, F). The mechanism by which TK exerts its protective effect, which curtails LPS-triggered inflammatory responses, delineates a novel therapeutic pathway for sepsis management.

### 3.7 TK modulation of intestinal microbiota homeostasis in LPS-induced septic mice

**FIGURE 8:**
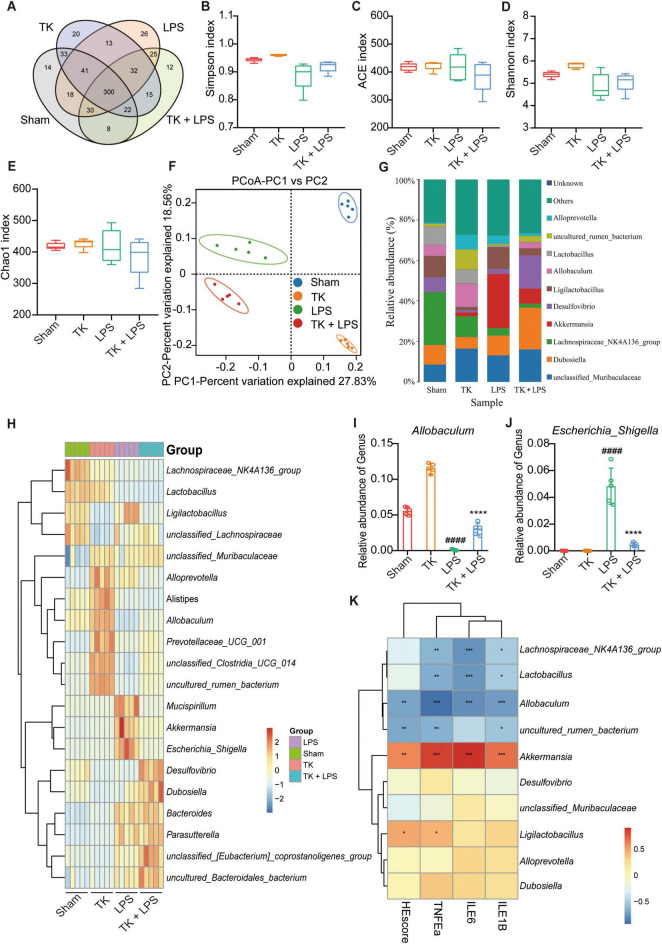
Effect of turmeric kombucha on the intestinal microbiota equilibrium in mice with LPS-induced sepsis. **(A)** Venn diagram showing overlapping operational taxonomic units (OTU) of intestinal microorganisms. **(B–E)** α-diversity of intestinal microbiota (OTU level): **(B)** Simpson index, **(C)** ACE index, **(D)** Shannon index, and **(E)** Chao1 index. **(F)** PCA plots based on weighted UniFrac Adonis analysis for different groups. **(G)** Stacked bar graph showing relative abundances at the genus level. **(H)** Heatmap of intestinal microorganism relative abundance across treatment groups at the genus level. **(I,J)** Relative abundances of *Allobaculum* **(I)** and *Escherichia*_*Shigella* **(J)**. **(K)** Spearman’s correlation heatmap: bacterial genera, HE scores, and IL-6, IL-1β, and TNF-α levels in mice with sepsis. The heatmap values represent the Z-values calculated by standardizing the relative abundance of the species in each row. The data analysis incorporated ANOVA and Tukey’s *post hoc* test (*n* = 5). ^####^*P* < 0.0001 against the sham group; ns, nonsignificant. *P < 0.05, **P < 0.01, ***P < 0.001, and *⁣*⁣**P < 0.0001 against the LPS group.

The sham, TK, LPS, and LPS + TK groups exhibited 14, 20, 26, and 12 unique operational taxonomic units (OTUs), respectively (Figure 8). α-diversity analysis revealed significant differences in the Shannon, Ace, Simpson, and Chao1 indices between the TK-treated and LPS groups (*P* < 0.01, Figures 8B-E). PCA revealed significant disparities in gut microbial communities among the groups (Figure 8F). We compared microbial community structure by analyzing the relative abundances at the phylum, family, and genus levels (Figure 8G and Supplementary Figures 3A, B). At the genus level, the dominant gut microbiota in mice included *Muribaculaceae*, *Dubosiella*, *Lachnospiraceae* NK4A136, *Akkermansia*, *Desulfovibrio*, *Ligilactobacillus*, *Allobaculum*, *Lactobacillus*, and *Alloprevotella* (Figure 8F). Figures 8G–K and Supplementary Figure 4 reveal that, among the LPS-induced mice, the relative abundances of *Alloprevotella*, *Lachnospiraceae* NK4A136 group, *Parabacteroides*, uncultured_bacterium_*Lachnospiraceae*, and uncultured_bacterium_*Muribaculaceae* were significantly higher in the TK-treated mice than in the non-TK mice (*P* < 0.05). Conversely, among the LPS-induced mice, the relative abundances of *Bacillus*, *Klebsiella*, and other genera were notably lower in the TK-treated group than in the non-TK group (*P* > 0.05).

Differences in the microbial community were analyzed using a LEfSe assay (Supplementary Figure 3C). Using LDA > 4.50 as the threshold, different species were identified (Supplementary Figure 3D). Subsequent correlation analysis was performed between the proinflammatory cytokines and intestinal microbiota. IL-1β, IL-6, and TNF-α expression in the serum and lung tissue was positively correlated with the abundance of *Akkermansia* and negatively correlated with the abundance of *Lachnospiraceae* NK4A136, *Lactobacillus*, and *Allobaculum* in the microbiota (Figure 8D). PICRUSt was used to predict functional genes in the LPS-induced mice with or without TK treatment; 28 significantly differentiated pathways were identified (Supplementary Figure 5). Together, these findings indicate that TK effectively reduced pathogenic bacterial abundance, increased beneficial bacterial abundance, and significantly ameliorated gut dysbiosis in mice with sepsis.

## 4 Discussion

Non-targeted metabolomics was used to analyze the compositional differences between TK and TW and to further investigate the protective effects and mechanisms of TK in mice with LPS-induced sepsis. Qualitative analysis identified 590 unique metabolites that differentiated TK from TW. Among the top 50 differential metabolites, maltulose and 1,4-beta-D-glucan exhibited the most notable differences. Future studies should consider using targeted metabolomic approaches to further validate and explore the mechanisms of action of TK.

**FIGURE 9:**
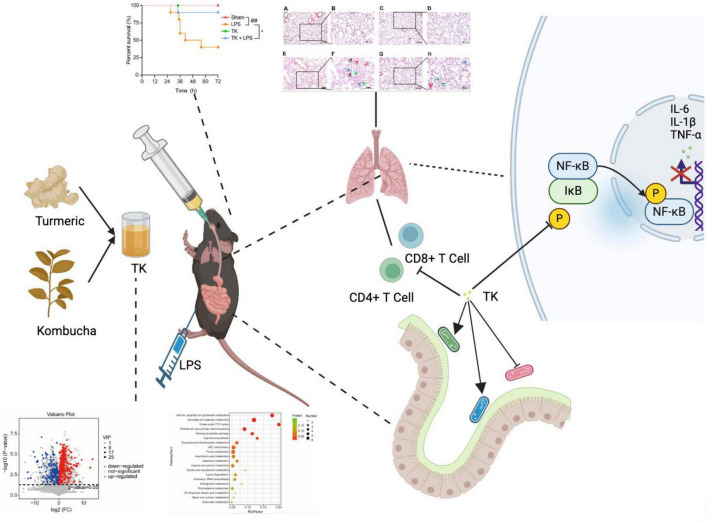
Turmeric kombucha (TK) ameliorates LPS-induced sepsis by attenuating inflammation and modulating gut microbiota.

As shown in Figure 9, TK significantly increased the survival rate of mice with LPS-induced sepsis, effectively improving their overall survival. It alleviated symptoms, such as lethargy, anorexia, and mobility reduction, while normalizing body temperature, ultimately increasing survival rates and quality of life. This demonstrates the preventative and therapeutic effects of TK in sepsis. TK markedly suppressed pro-inflammatory cytokine expression (IL-6, TNF-α, and IL-1β) in the LPS-induced mice, reducing the inflammatory burden and modulating immune responses. Furthermore, TK reduced activated CD4+ T and CD8+ T cell counts, attenuated excessive immune responses, and downregulated critical proteins in the NF-κB pathway. In the LPS-induced septic mice, TK induced significant changes in the mouse gut microbiota, notably increasing *Lachnospiraceae* NK4A136, *Lactobacillus*, and *Allobaculum*, which are associated with reduced inflammatory cytokine expression.

Kombucha has gained prominence owing to its health benefits, including its ability to reduce inflammation. Turmeric, which is rich in bioactive compounds, such as polyphenols and curcumin, is traditionally used as a spice or coloring agent. Curcumin, extracted from turmeric rhizomes, exhibits antioxidant, anti-inflammatory, and anticancer properties (Fadus et al., 2017).

During sepsis, monocytes and neutrophils release a wide array of pro-inflammatory cytokines, leading to a significant increase in lymphocyte numbers and triggering a “cytokine storm,” which is associated with cellular toxicity (Weber et al., 2015). The introduction of cells undergoing programmed cell death substantially mitigates this “cytokine storm” (Karbian et al., 2020). Consequently, reducing inflammation and apoptosis is essential for managing sepsis (Su et al., 2023c,2023d,2024a; 2024b; 2024c). The anti-inflammatory effects of TK in mice with LPS-induced sepsis may be linked to curcumin. Curcumin modulates several cell-signaling pathways, leading to the downregulation of pro-inflammatory adipokines and the upregulation of anti-inflammatory gene products.

The anti-inflammatory properties of kombucha are attributed to its beneficial constituents, such as flavonoids and phenolic acids (gallic acid, catechins, and theaflavins), which are produced via biotransformation and metabolism by the flora in the kombucha. The phenolic content of kombucha from oak-leaf tea fermentation was found to contribute to its pronounced anti-inflammatory and antioxidant effects (Vázquez-Cabral et al., 2017). Similarly, the inhibitory activities of phenolic compounds from peony flowers on bacterial biofilm formation highlight the potential for phenolics in modulating microbiota and inflammation, akin to the phenolic content in TK (Li C. et al., 2020). Additionally, the influence of different extraction methods on the antibacterial and antioxidant activities of phenolic compounds from areca nuts emphasizes the importance of optimizing extraction processes to enhance the therapeutic potential of bioactive compounds (Fan et al., 2022). Using yerba mate extract as an alternative fermentation substrate produced kombucha that significantly inhibited lipoxygenase activity after 14 and 21 days of fermentation (Ziemlewska et al., 2021). The development of herbal extract-based compounds for enhancing immunity and protecting gastric mucosa underscores the relevance of combining bioactive compounds for therapeutic purposes. This aligns with our observation of the capacity of TK to improve survival rates and reduce inflammation in septic mice (Pei et al., 2021).

Quercetin and saponins, the primary anti-inflammatory agents in yerba mate extract, reduce the production of IL-6, COX-2, and NO, the main inflammatory mediators. The tannins in yerba mate extract inhibit the activity of LOX (Ziemlewska et al., 2021), which is integral to the synthesis of leukotriene, a key mediator of the pro-inflammatory response; regulation of LOX is therefore crucial for treating inflammation (Oguntibeju, 2018). Zedoarondiol in LPS-stimulated mouse macrophages markedly suppresses the NF-κB pathway, thus diminishing iNOS, COX-2, and pro-inflammatory cytokine production (Cho et al., 2009).

Although few studies have examined the anti-inflammatory effects of TK, TK has been demonstrated to be an effective immunomodulator (Su et al., 2023e; Zubaidah et al., 2021). The pathogenesis of sepsis involves lymphocyte replication, apoptosis induction, and the upregulation of anti-inflammatory molecules, co-suppressor receptors, and ligands (van der Poll et al., 2017; Venet and Monneret, 2018). The investigation into mechanisms and ingredients promoting the formation of beneficial bacterial biofilm provides insights into the microbiota-modulating effects observed with TK, further supporting its therapeutic potential (Lou et al., 2023).

During sepsis, neutrophils interact with endothelial cells, migrate to sites of inflammation, phagocytose pathogens, and release active factors and enzymes (Shen et al., 2017). Upon cytokine stimulation or pathogen exposure, mononuclear cells or macrophages phagocytose pathogens and present antigens. Effector T cells activate macrophages, which secrete mediators that produce fibrotic tissue (Hou et al., 2015). Dendritic cell (DC) maturation in the spleen and lymph nodes is impeded during sepsis, and DC activation leads to the rapid accumulation of innate immune cells, including monocytes, NK cells, and granulocytes. In patients with sepsis, defective monocyte metabolism occurs during immunosuppression, which is characterized by inhibited glycolysis, fatty acid oxidation, and oxidative phosphorylation (Cheng et al., 2016). In sepsis, NK cells produce high levels of INF-γ, but lose the ability to support the Th1 immune responses necessary for bacterial infection clearance (Guo et al., 2018). Sepsis survivors may thus develop immunosuppression, failing to clear primary infections and becoming susceptible to secondary infections and viral reactivation. This immunosuppression, which involves both innate and adaptive immunity, is regulated by co-stimulatory molecules, such as CD80/B7-1, that are produced via TLR pathway activation and cytokine-induced regulatory T cell transformation; this in turn reduces the expression of antigen presentation-related transcription factors (Pepin et al., 2018).

In this study, TK demonstrated strong anti-inflammatory effects in an acute lung injury model. It significantly improved lung tissue condition in mice, reducing inflammation and immune cell infiltration. LPS stimulation induces cytokines that drive T- and B-lymphocyte differentiation, triggering the activation of cellular immunity (Gao et al., 2020). Additionally, the multifunctional biological potential of cyclic lipopeptides like Iturin, known for their antibacterial and immune-modulating properties, parallels the broad-spectrum impact observed under TK treatment (Wan et al., 2022). However, among the LPS-induced mice, the TK-treated group exhibited markedly lower CD4+ T and CD8+ T cell activation than that of the non-TK group, revealing the modulatory effect of TK on the immune response.

The intestinal microbiota significantly affects overall health, and its metabolic activity affects the host via the gut–brain axis, modifying appetite, food consumption, energy balance, glucose and insulin metabolism, and lipid status (Costa et al., 2023). Dysbiosis of the gut microbiome is associated with increased intestinal permeability, allowing the migration of certain microbes and their products, particularly LPS, into the bloodstream. This process activates TLR-4-mediated immune responses, exacerbating the inherent inflammatory cascades of obesity (Gasmi Benahmed et al., 2021). Via the endotoxin pathway, gut microbiota dysbiosis, which involves an increase in gram-negative bacteria or pathogens and a reduction in beneficial bacteria, disrupts the intestinal mucosal barrier by increasing intestinal LPS levels (Li Y. et al., 2020). Pretreatment with TK significantly affects the intestinal flora of mice, improving their microbiotic diversity and composition and increasing the production of probiotics that maintain intestinal homeostasis (Paul et al., 2023).

Based on our findings, the community structure of the intestinal flora was disrupted under LPS-induced sepsis, exacerbating the damage caused by sepsis. TK intervention protected intestinal homeostasis, with *Dubosiella* being the dominant bacterial genus in the TK-treated LPS group. Tk thus plays a potentially vital role in obesity treatment and the maintenance of intestinal balance. Verrucomicrobiota were significantly more abundant in the non-TK LPS group than in the TK-treated LPS and sham groups. However, after TK treatment, the abundance of Verrucomicrobiota decreased in the LPS group. The Verrucomicrobiota, which typically constitutes approximately 0.1% of the gut microbiota, includes genera such as Verrucomicrobium and Pedosphaera. Species-level analysis of the mouse gut microbiota revealed that the most significant increase in Verrucomicrobiota was in *Akkermansia muciniphila*, one of the most abundant members of the human gut microbiome, accounting for 1–5% (Derrien et al., 2008); this anaerobic gut bacterium is crucial in maintaining intestinal homeostasis (Bae et al., 2022). Its unique function in inducing adaptive immune responses in the gut during T-cell subset homeostasis makes these correlations even more valuable (Bae et al., 2022). The immunogenicity of *A. muciniphila* stems from the lipids on its cell membrane, specifically the active molecule 12-methyltetradecanoyl-13-methylmyristoyl-sn-glycero-3-phosphoethanolamine (standard lipid nomenclature: a15:0-i15:0 PE), which can induce DC cells to produce TNF-α and IL-6 without secreting IL-10 and IL-27P10. This potent phosphatidylethanolamine is not commonly found in the normal microbiota and has not been identified in immune-reactive gut bacteria (Depommier et al., 2019). Here, in the TK group, but not in LPS-induced sepsis, the presence of *A. muciniphila* and the reduction in the Firmicutes/Bacteroidetes ratio suggest that TK alters the gut microbial community structure. Among the LPS-induced septic mice, *A. muciniphila* abundance was substantially elevated in the non-TK group, but was lower in the TK-treated group. Furthermore, the *A. muciniphila* lipid component a15:0-i15:0 PE can intensify the LPS-induced inflammatory response (Pepin et al., 2018), suggesting that *A. muciniphila* enhances LPS stimulation.

## 5 Conclusion

This study underscores the protective effects of TK against LPS-induced sepsis and its impact on the intestinal microbiota of mice. Using TW as a control, we employed non-targeted metabolomics to analyze the compositional differences between TK and TW, identifying 590 unique metabolites. TK significantly improved survival rates and quality of life in mice with LPS-induced sepsis by alleviating symptoms and normalizing body temperature. TK exerts both preventative and therapeutic effects in sepsis by markedly suppressing pro-inflammatory cytokine expression, modulating immune responses, and reducing excessive activation of CD4^+^ and CD8^+^ T cells. Additionally, TK beneficially alters gut microbiota composition, increasing bacterial genera associated with reduced inflammation, thereby further contributing to its anti-inflammatory effects. Future research should utilize targeted metabolomics to validate these findings and explore specific mechanisms, paving the way for targeted sepsis therapies and expanding kombucha applications.

## Data Availability

All data included in this study are available upon request by contact with the corresponding authors.

## Footnotes

1
https://beikolab.cs.dal.ca/software

## References

[B1] Bae M. Cassilly C. D. Liu X. Park S. M. Tusi B. K. Chen X. (2022). *Akkermansia muciniphila* phospholipid induces homeostatic immune responses. *Nature* 608 168–173. 10.1038/s42003-024-05867-6 35896748 PMC9328018

[B2] Cardoso R. R. Moreira L. P. D. de Campos Costa M. A. Toledo R. C. L. Grancieri M. Nascimento T. P. D. (2021). Kombuchas from green and black teas reduce oxidative stress, liver steatosis and inflammation, and improve glucose metabolism in Wistar rats fed a high-fat high-fructose diet. *Food Funct.* 12 10813–10827. 10.1039/d1fo02106k 34617537

[B3] Cheng S. C. Scicluna B. P. Arts R. J. Gresnigt M. S. Lachmandas E. Giamarellos-Bourboulis E. J. (2016). Broad defects in the energy metabolism of leukocytes underlie immunoparalysis in sepsis. *Nat. Immunol.* 17 406–413. 10.1038/ni.3398 26950237

[B4] Cho W. Nam J. W. Kang H. J. Windono T. Seo E. K. Lee K. T. (2009). Zedoarondiol isolated from the rhizoma of *Curcuma heyneana* is involved in the inhibition of iNOS, COX-2 and pro-inflammatory cytokines via the downregulation of NF-kappaB pathway in LPS-stimulated murine macrophages. *Int. Immunopharmacol.* 9 1049–1057. 10.1016/j.intimp.2009.04.012 19398040

[B5] Costa M. A. C. Vilela D. L. S. Fraiz G. M. Lopes I. L. Coelho A. I. M. Castro L. C. V. (2023). Effect of kombucha intake on the gut microbiota and obesity-related comorbidities: A systematic review. *Crit. Rev. Food Sci. Nutr.* 63 3851–3866. 10.1080/10408398.2021.1995321 34698580

[B6] Depommier C. Everard A. Druart C. Plovier H. Van Hul M. Vieira-Silva S. (2019). Supplementation with *Akkermansia muciniphila* in overweight and obese human volunteers: A proof-of-concept exploratory study. *Nat. Med.* 25 1096–1103. 10.1038/s41591-019-0495-2 31263284 PMC6699990

[B7] Derrien M. Collado M. C. Ben-Amor K. Salminen S. de Vos W. M. (2008). The Mucin degrader *Akkermansia muciniphila* is an abundant resident of the human intestinal tract. *Appl. Environ. Microbiol.* 74 1646–1648. 10.1128/AEM.01226-07 18083887 PMC2258631

[B8] Fadus M. C. Lau C. Bikhchandani J. Lynch H. T. (2017). Curcumin: An age-old anti-inflammatory and anti-neoplastic agent. *J. Tradit. Complement. Med.* 7 339–346. 10.1016/j.jtcme.2016.08.002 28725630 PMC5506636

[B9] Fan X. Jiang C. Dai W. Jing H. Du X. Peng M. (2022). Effects of different extraction on the antibacterial and antioxidant activities of phenolic compounds of areca nut (husks and seeds). *J Food Meas Charact.* 16 1502–1515. 10.1007/s11694-021-01244-7

[B10] Gao Y. Jin H. Tan H. Wang Y. Wu J. Wang Y. (2020). The role of extracellular vesicles from stored RBC units in B lymphocyte survival and plasma cell differentiation. *J. Leukoc. Biol.* 108 1765–1776. 10.1002/jlb.1a0220-666r 32421907

[B11] Gasmi Benahmed A. Gasmi A. Doşa A. Chirumbolo S. Mujawdiya P. K. Aaseth J. (2021). Association between the gut and oral microbiome with obesity. *Anaerobe* 70:102248. 10.1016/j.anaerobe.2020.102248 32805390

[B12] Guo Y. Patil N. K. Luan L. Bohannon J. K. Sherwood E. R. (2018). The biology of natural killer cells during sepsis. *Immunology* 153 190–202. 10.1111/imm.12854 29064085 PMC5765373

[B13] Hou J. Chen Q. Zhang K. Cheng B. Xie G. Wu X. (2015). Sphingosine 1-phosphate receptor 2 signaling suppresses macrophage phagocytosis and impairs host defense against sepsis. *Anesthesiology* 123 409–422. 10.1097/aln.0000000000000725 26200183

[B14] Huang M. Cai S. Su J. (2019). The pathogenesis of sepsis and potential therapeutic targets. *Int. J. Mol. Sci.* 20:5376. 10.3390/ijms20215376 31671729 PMC6862039

[B15] Karbian N. Abutbul A. El-Amore R. Eliaz R. Beeri R. Reicher B. (2020). Apoptotic cell therapy for cytokine storm associated with acute severe sepsis. *Cell Death Dis.* 117:535. 10.1038/s41419-020-02748-8 32669536 PMC7363887

[B16] Karimian M. S. Pirro M. Majeed M. Sahebkar A. (2017). Curcumin as a natural regulator of monocyte chemoattractant protein-1. *Cytokine Growth Factor Rev.* 33 55–63. 10.1016/j.cytogfr.2016.10.001 27743775

[B17] Li C. Jiang C. Jing H. Jiang C. Wang H. Du X. (2020). Separation of phenolics from peony flowers and their inhibitory activities and action mechanism on bacterial biofilm. *Appl. Microbiol. Biotechnol.* 104 4321–4332. 10.1007/s00253-020-10540-z 32232531

[B18] Li L. Wang L. Fan W. Jiang Y. Zhang C. Li J. (2020). The application of fermentation technology in traditional Chinese medicine: A review. *Am. J. Chin. Med.* 484 899–921. 10.1142/s0192415x20500433 32431179

[B19] Li Y. Rahman S. U. Huang Y. Zhang Y. Ming P. Zhu L. (2020). Green tea polyphenols decrease weight gain, ameliorate alteration of gut microbiota, and mitigate intestinal inflammation in canines with high-fat-diet-induced obesity. *J. Nutr. Biochem.* 78:108324. 10.1016/j.jnutbio.2019.108324 32004926

[B20] Lou Z. Zheng X. Bede D. Dai W. Wan C. Wang H. (2023). New perspectives for mechanisms, ingredients, and their preparation for promoting the formation of beneficial bacterial biofilm. *J. Food Meas. Charact.* 17 2386–2403. 10.1007/s11694-022-01777-5

[B21] Mousavi S. M. Hashemi S. A. Zarei M. Gholami A. Lai C. W. Chiang W. H. (2020). Recent progress in chemical composition, production, and pharmaceutical effects of kombucha beverage: A complementary and alternative medicine. *Evid. Based Complement. Alternat. Med.* 2020:4397543. 10.1155/2020/4397543 33281911 PMC7688354

[B22] Oguntibeju O. O. (2018). Medicinal plants with anti-inflammatory activities from selected countries and regions of Africa. *J. Inflamm. Res.* 11 307–317. 10.2147/JIR.S167789 30122972 PMC6086115

[B23] Paul A. K. Lim C. L. Apu M. A. I. Dolma K. G. Gupta M. de Lourdes Pereira M. (2023). Are fermented foods effective against inflammatory diseases. *Int. J. Environ. Res. Public Health* 20:2481. 10.3390/ijerph20032481 36767847 PMC9915096

[B24] Pei Z. Lou Z. Zhang B. Wang H. Li Y. (2021). Development of a compound oral liquid containing herbal extracts and its effect on immunity and gastric mucosa. *J. Food Sci.* 86 2684–2699. 10.1111/1750-3841.15761 34096062

[B25] Pepin D. Godeny M. Russell D. Mehta P. Lie W. (2018). Profiling of soluble immune checkpoint proteins as potential non-invasive biomarkers in colorectal cancer and sepsis. *J. Immunol.* 200:174.43. 10.4049/jimmunol.200.Supp.174.43

[B26] Qu L. Li Y. Chen C. Yin T. Fang Q. Zhao Y. (2022). Caveolin-1 identified as a key mediator of acute lung injury using bioinformatics and functional research. *Cell Death Dis.* 13:686. 10.1038/s41419-022-05134-8 35933468 PMC9357074

[B27] Russell J. A. (2006). Management of sepsis. *N. Engl. J. Med.* 355 1699–1713. 10.1056/nejmra043632 17050894

[B28] Shen X. F. Cao K. Jiang J. P. Guan W. X. Du J. F. (2017). Neutrophil dysregulation during sepsis: An overview and update. *J. Cell Mol. Med.* 21 1687–1697. 10.1111/jcmm.13112 28244690 PMC5571534

[B29] Shrum B. Anantha R. V. Xu S. X. Donnelly M. Haeryfar S. M. McCormick J. K. (2014). A robust scoring system to evaluate sepsis severity in an animal model. *BMC Res. Notes* 7:233. 10.1186/1756-0500-7-233 24725742 PMC4022086

[B30] Singer M. Deutschman C. S. Seymour C. W. Shankar-Hari M. Annane D. Bauer M. (2016). The third international consensus definitions for sepsis and septic shock (Sepsis-3). *JAMA* 315 801–810. 10.1001/jama.2016.0287 26903338 PMC4968574

[B31] Su J. Chen W. Zhou F. Li R. Tong Z. Wu S. (2024a). Inhibitory mechanisms of decoy receptor 3 in cecal ligation and puncture-induced sepsis. *mBio* 15:e0052124. 10.1128/mbio.00521-24 38700314 PMC11237498

[B32] Su J. Tong Z. Feng Z. Wu S. Zhou F. Li R. (2024b). Protective effects of DcR3-SUMO on lipopolysaccharide-induced inflammatory cells and septic mice. *Int. J. Biol. Macromol.* 275:133703. 10.1016/j.ijbiomac.2024.133703 38986982

[B33] Su J. Xiao J. Chen S. Zhao H. Zhang X. Feng Z. (2024c). Aloin ameliorates cecal ligation and puncture-induced sepsis in mice by attenuating inflammation and modulating gut microbiota. *Food Sci. Hum. Wellness* 10.26599/FSHW.2024.9250034

[B34] Su J. Tan Q. Tang Q. Tong Z. Yang M. (2023b). Research progress on alternative kombucha substrate transformation and the resulting active components. *Front. Microbiol.* 14:1254014. 10.3389/fmicb.2023.1254014 37779696 PMC10537971

[B35] Su J. Zhou F. Wu S. Tong Z. (2023e). Research progress on natural small-molecule compounds for the prevention and treatment of sepsis. *Int. J. Mol. Sci.* 24:12732. 10.3390/ijms241612732 37628912 PMC10454676

[B36] Su J. Guan B. Chen K. Feng Z. Guo K. Wang X. (2023a). Fucoxanthin attenuates inflammation via interferon regulatory factor 3 (IRF3) to improve sepsis. *J. Agric. Food Chem.* 71 12497–12510. 10.1021/acs.jafc.3c03247 37560933

[B37] Su J. Tan Q. Wu S. Abbas B. Yang M. (2023c). Application of kombucha fermentation broth for antibacterial, antioxidant, and anti-inflammatory processes. *Int. J. Mol. Sci.* 24:13984. 10.3390/ijms241813984 37762292 PMC10530541

[B38] Su J. Wu S. Zhou F. Tong Z. (2023d). Research progress of macromolecules in the prevention and treatment of sepsis. *Int. J. Mol. Sci.* 24:3017. 10.3390/ijms241613017 37629199 PMC10455590

[B39] Tran T. Grandvalet C. Verdier F. Martin A. Alexandre H. Tourdot-Maréchal R. (2020). Microbiological and technological parameters impacting the chemical composition and sensory quality of kombucha. *Compr. Rev. Food Sci. Food Saf.* 19 2050–2070. 10.1111/1541-4337.12574 33337078

[B40] van der Poll T. van de Veerdonk F. L. Scicluna B. P. Netea M. G. (2017). The immunopathology of sepsis and potential therapeutic targets. *Nat. Rev. Immunol.* 17 407–420. 10.1038/nri.2017.36 28436424

[B41] Vázquez-Cabral B. D. Larrosa-Pérez M. Gallegos-Infante J. A. Moreno-Jiménez M. R. González-Laredo R. F. Rutiaga-Quiñones J. G. (2017). Oak kombucha protects against oxidative stress and inflammatory processes. *Chem. Biol. Interact.* 272 1–9. 10.1016/j.cbi.2017.05.001 28476604

[B42] Venet F. Monneret G. (2018). Advances in the understanding and treatment of sepsis-induced immunosuppression. *Nat. Rev. Nephrol.* 14 121–137. 10.1038/nrneph.2017.165 29225343

[B43] Wan C. Fan X. Lou Z. Wang H. Olatunde A. Rengasamy K. R. R. (2022). Iturin: Cyclic lipopeptide with multifunction biological potential. *Crit. Rev. Food Sci. Nutr.* 62 7976–7988. 10.1080/10408398.2021.1922355 33983074

[B44] Wang M. Chen G. Chen D. Ye H. Sun Y. Zeng X. (2019). Purified fraction of polysaccharides from Fuzhuan brick tea modulates the composition and metabolism of gut microbiota in anaerobic fermentation in vitro. *Int. J. Biol. Macromol.* 140 858–870. 10.1016/j.ijbiomac.2019.08.187 31446105

[B45] Wang P. Zhang H. Liu B. Yang M. (2021b). Interaction of strains in kombucha microbial community affects their growth and metabolism. *Microbiol. China* 48 426–436. 10.13344/j.microbiol.china.200299

[B46] Wang P. Feng Z. Sang X. Chen W. Zhang X. Xiao J. (2021a). Kombucha ameliorates LPS-induced sepsis in a mouse model. *Food Funct.* 12 10263–10280. 10.1039/D1FO01839F 34549751

[B47] Weber G. F. Chousterman B. G. He S. Fenn A. M. Nairz M. Anzai A. (2015). Interleukin-3 amplifies acute inflammation and is a potential therapeutic target in sepsis. *Science* 347 1260–1265. 10.1126/science.aaa4268 25766237 PMC4376966

[B48] Yang Z. J. Huang S. Y. Zhou D. D. Xiong R. G. Zhao C. N. Fang A. P. (2022). Effects and mechanisms of curcumin for the prevention and management of cancers: An updated review. *Antioxidants (Basel)* 11:1481. 10.3390/antiox11081481 36009200 PMC9405286

[B49] Zhang C. Zhao M. Wang B. Su Z. Guo B. Qin L. (2021). The Nrf2-NLRP3-caspase-1 axis mediates the neuroprotective effects of Celastrol in Parkinson’s disease. *Redox Biol.* 47:102134. 10.1016/j.redox.2021.102134 34600334 PMC8487081

[B50] Ziemlewska A. Nizioł-Łukaszewska Z. Bujak T. Zagórska-Dziok M. Wójciak M. Sowa I. (2021). Effect of fermentation time on the content of bioactive compounds with cosmetic and dermatological properties in Kombucha Yerba Mate extracts. *Sci. Rep.* 11:18792. 10.1038/s41598-021-98191-6 34552134 PMC8458284

[B51] Zubaidah E. Nisak Y. K. Susanti I. Widyaningsih T. D. Srianta I. Tewfik I. (2021). Turmeric Kombucha as effective immunomodulator in *Salmonella typhi*-infected experimental animals. *Biocatal. Agric. Biotechnol.* 37:102181. 10.3390/foods12091818 37174355 PMC10178031

