# Supplementary material for: Administration of turmeric kombucha ameliorates lipopolysaccharide-induced sepsis by attenuating inflammation and modulating gut microbiota
Source: Front Microbiol. 2024 Aug 30;15:1452190. doi: 10.3389/fmicb.2024.1452190 (PMC11392888; doi:10.3389/fmicb.2024.1452190)
Supplement: Supplementary file 1 [file Table_1.DOCX]

**Supplementary figures for**

**Administration of Turmeric Kombucha Ameliorates Lipopolysaccharide-Induced Sepsis by Attenuating Inflammation and Modulating Gut Microbiota**

Jingqian Su ^†, *^, Qingqing Tan ^†^, Shun Wu, Fen Zhou, Chen Xu, Heng Zhao, Congfan Lin, Xiaohui Deng, Lian Xie, Xinrui Lin, Hui Ye, Minhe Yang ^*^

Fujian Key Laboratory of Innate Immune Biology, Biomedical Research Center of South China, College of Life Science, Fujian Normal University, Fuzhou, Fujian Province 350117, PR China.

*Correspondence: [sjq027@fjnu.edu.cn](mailto:sjq027@fjnu.edu.cn) (J. Su); [minhe214@fjnu.edu.cn](mailto:minhe214@fjnu.edu.cn) (M. Yang)

† These authors contributed equally to this work.


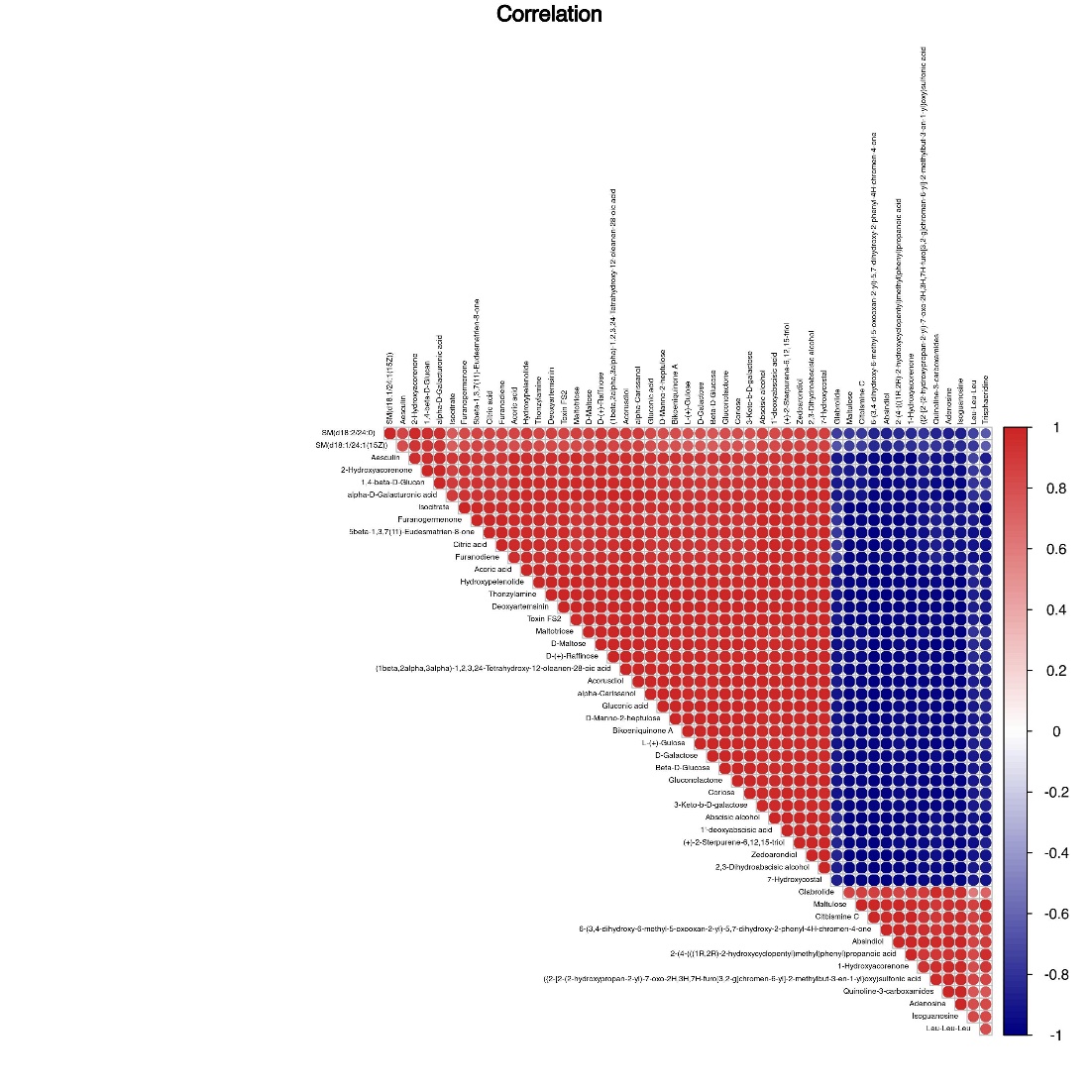


**Fig. S1. Correlation analysis of TOP-50 differential metabolites.** Note: Red indicates positive correlation, blue indicates negative correlation.


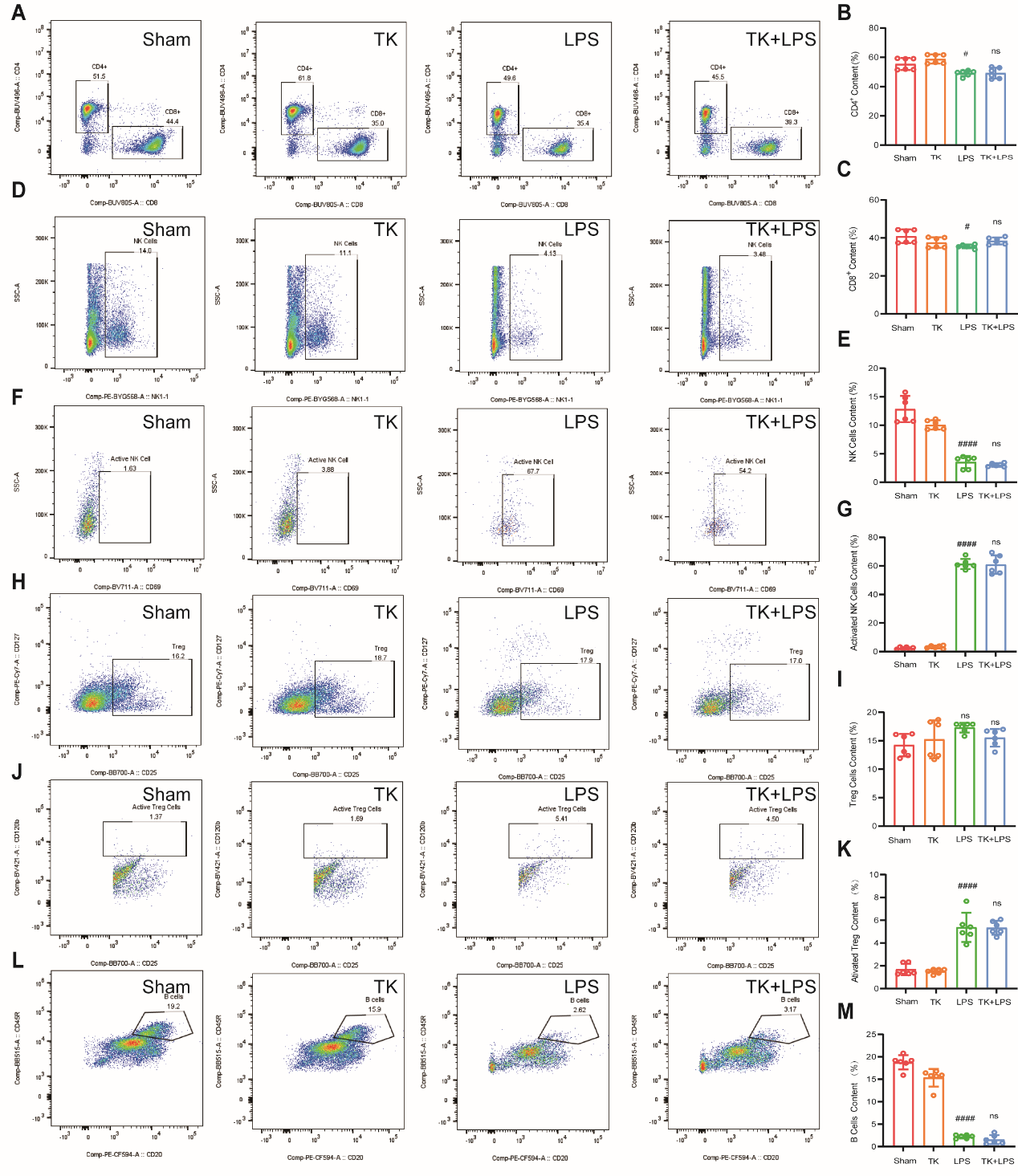

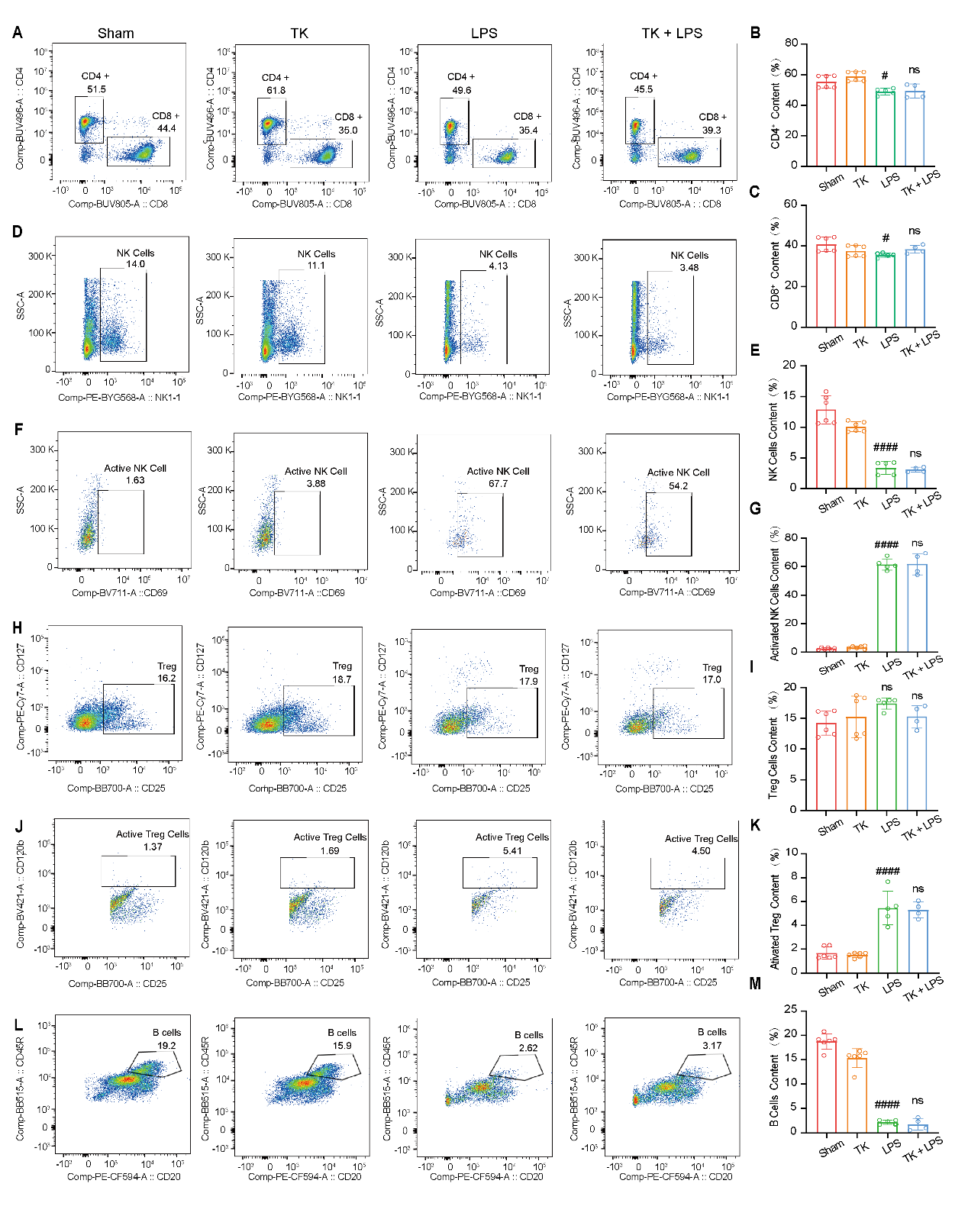


**Fig. S2. Effects of TK treatments on the expression of CD4^+^ T, CD8^+^ T, NK, activated NK, Treg, activated** **Treg and B cells in peripheral blood of LPS-induced sepsis mice.** (A) Flow chart and detection markers for CD4^+^ T and CD8^+^ T assessed using flow cytometry in LPS-induced sepsis mice with the TK treatment. (B) Number of CD4^+^ T cells. (C) Number of CD8^+^ T cells. (D) Flow chart and detection markers for NK assessed using flow cytometry in LPS-induced sepsis mice with the TK treatment. (E) Number of NK cells. (F) Flow chart and detection markers for activated NK cells assessed using flow cytometry in LPS-induced sepsis mice with the TK cells treatment. (G) Number of activated NK cells. (H) Flow chart and detection markers for activated Treg cells assessed using flow cytometry in LPS-induced sepsis mice with the TK treatment. (I) Number of activated Treg cells. (J) Flow chart and detection markers for activated Treg cells assessed using flow cytometry in LPS-induced sepsis mice with the TK treatment. (K) Number of activated activated Treg cells. (L) Flow chart and detection markers for activated B cells assessed using flow cytometry in LPS-induced sepsis mice with the TK treatment. (M) Number of activated B cells. ANOVA and Tukey’s post hoc test were performed to analyze the data (*n* = 5). (#) *p <* 0.05 and (####) *p <* 0.0001 vs. the sham group; (ns) *p >* 0.05 *vs* the LPS group.


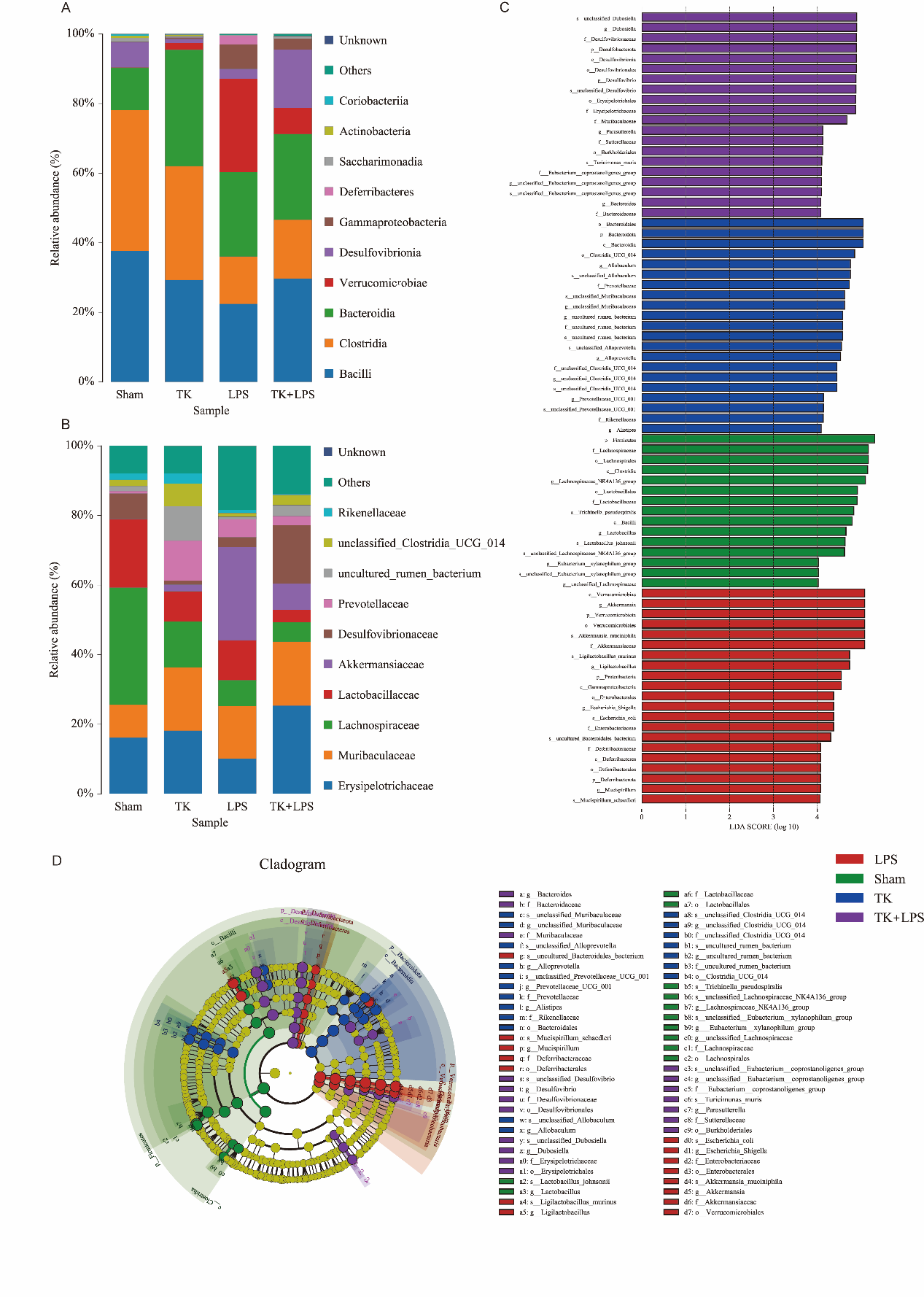
 **Fig. S3. Impact of TK on fecal microbiota across mouse groups.** (A-B) Gut microbiota composition: class level (A) and family level (B). (C) LEfSe analysis of the gut microbiota: LDA score > ± 4.0. (D) Cladogram derived from lefse analysis.


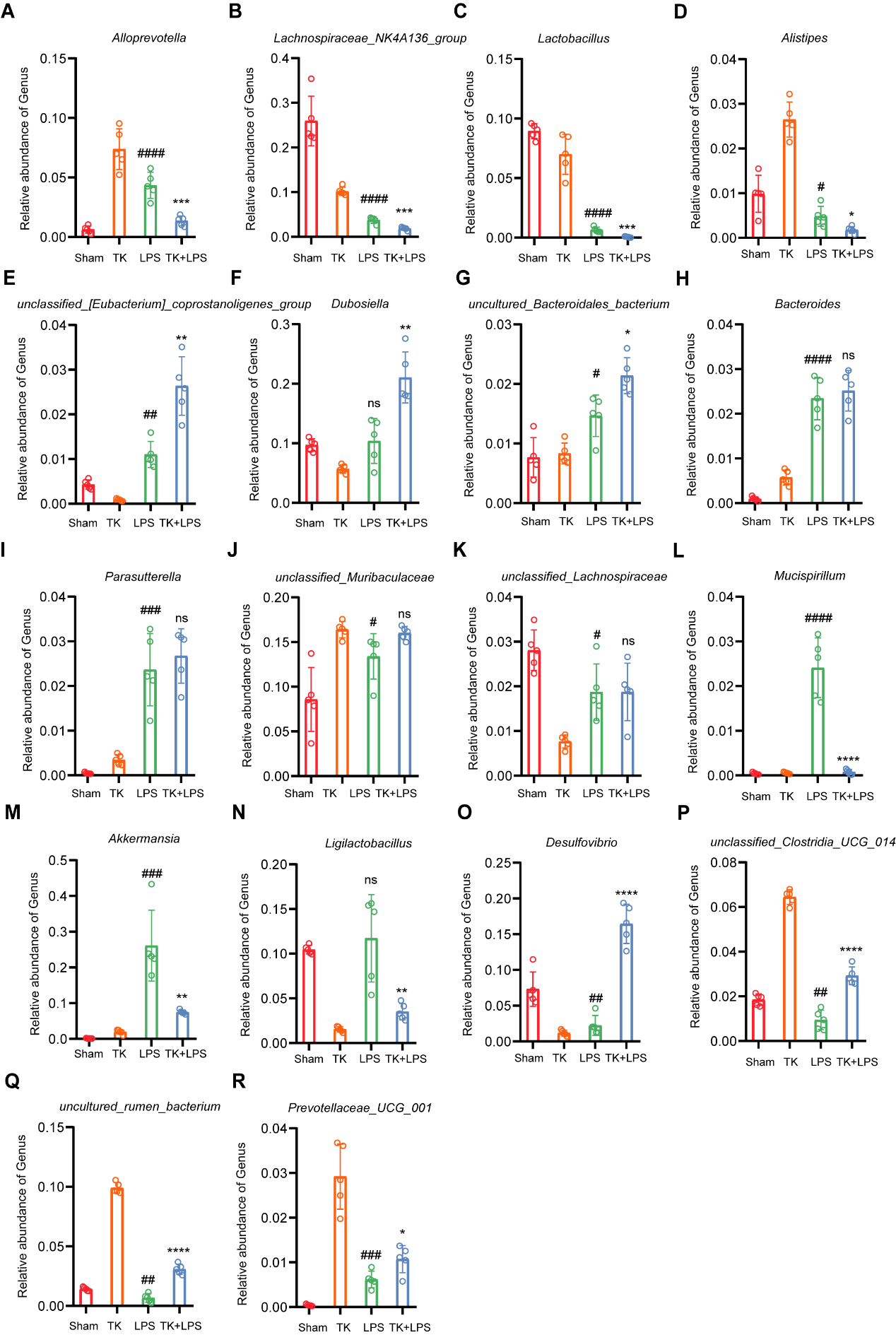


**Fig. S4. Relative abundance of intestinal microorganisms in different groups at the genus level.** (A) Relative abundance of *Alloprevotella*, (B) *Lachnospiraceae*_NK4A136_group, (C) *Lactobacillus,* (D) *Alistipes,* (E) *unclassified_[Eubacterium]_coprostanoligenes*_group, (F) *Dubosiella*, (G) uncultured_Bacteroidales_bacterium, (H) *Bacteroides*, (I) *Parasutterella*, (J) *unclassified_Muribaculaceae,* (K) *unclassified_Lachnospiraceae*, (L) *Mucispirillum*, (M) *Akkermansia*, (N) *Ligilactobacillus*, (O) *Desulfovibrio*, (P) *unclassified_Clostridia_UCG_014*, (Q) *uncultured_rumen_bacterium*, (R) *Prevotellaceae_UCG_001.* ANOVA and Tukey’s post hoc test were performed to analyze the data (*n* = 5). (#) *p <* 0.05, (##) *p <* 0.01, (###) *p <* 0.001 and (####) *p <* 0.0001 vs. the sham group; (ns) *p >* 0.05, (*) *p <* 0.05, (**) *p <* 0.01, and (***) *p <* 0.001 *vs* the LPS group.


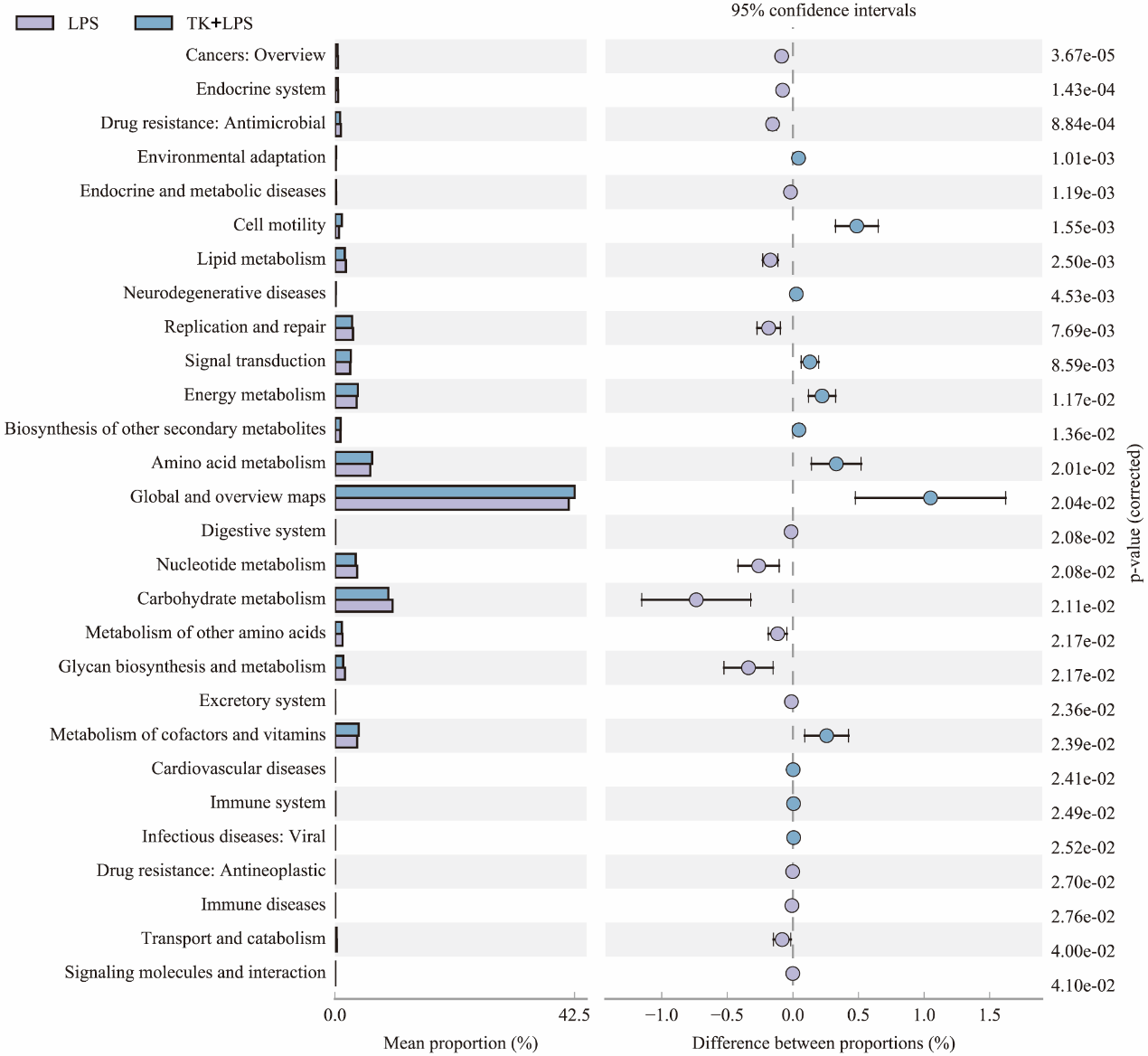


**Fig. S5. PICRUSt function prediction of the fecal microbiota in LPS and TK + LPS groups.** Purple bar, LPS group; Blue bar, TK + LPS group.

**Table S1 Statistical table of material classification distribution of TW/TK differential metabolites**

| **Material Classification** | **Number** | **Proportion** | **Up-Regulation Number** | **Up-Proportion** | **Down-Number** | **Down-Proportion** |
| --- | --- | --- | --- | --- | --- | --- |
| Lipids and Lipid-like Molecules | 280 | 47.46% | 198 | 70.71% | 82 | 29.29% |
| Oxygen-containing Organic Compounds | 73 | 12.37% | 60 | 82.19% | 13 | 17.81% |
| Organic Acids and Derivatives | 38 | 6.44% | 27 | 71.05% | 11 | 28.95% |
| Aromatic Compounds | 36 | 6.10% | 26 | 72.22% | 10 | 27.78% |
| Phenylpropanoids and Polyketides | 30 | 5.08% | 18 | 60.00% | 12 | 40.00% |
| Organic Heterocyclic Compounds | 28 | 4.75% | 19 | 67.86% | 9 | 32.14% |
| Nucleosides, Nucleotides, and Analogues | 11 | 1.86% | 2 | 18.18% | 9 | 81.82% |
| Organic Sulfur Compounds | 2 | 0.34% | - | 0.00% | - | 0.00% |
| Nitrogen-containing Heterocyclic Compounds | 2 | 0.34% | 2 | 100.00% | 2 | 100.00% |
| Hydrocarbon Derivatives | 1 | 0.17% | 1 | 100.00% | - | 0.00% |
| Alkaloids and Derivatives | 1 | 0.17% | 1 | 100.00% | - | 0.00% |
| Hydrocarbons | 1 | 0.17% | - | 0.00% | 1 | 100.00% |
| Unclassified | 87 | 14.75% | 60 | 68.97% | 27 | 31.03% |

**Table S2 TK/TW carbohydrate and analogues differential metabolites**

| **m/z** | **Retention time(min)** | **Metabolites** | **Molecular formula** | **VIP** | ***P* value** | **FC** | **log_2_(FC)** |
| --- | --- | --- | --- | --- | --- | --- | --- |
| 195.050 | 0.708 | Gluconic Acid | C_6_H_12_O_7_ | 22.02 | 0.000 | 12.19 | 3.61 |
| 381.079 | 0.748 | D-Maltose | C_12_H_22_O_11_ | 19.96 | 0.000 | 1.32 | 0.40 |
| 193.035 | 0.708 | α-D-Galacturonic Acid | C_6_H_10_O_7_ | 15.48 | 0.001 | 18.38 | 4.20 |
| 543.132 | 0.748 | Maltotriose | C_18_H_32_O_16_ | 11.89 | 0.000 | 1.72 | 0.78 |
| 203.053 | 0.712 | L-(+)-Glucose | C_6_H_12_O_6_ | 8.57 | 0.000 | 3.23 | 1.69 |
| 223.045 | 0.764 | Gluconolactone | C_6_H_10_O_6_ | 5.80 | 0.000 | 3.29 | 1.72 |
| 549.167 | 0.764 | D-(+)-Gossypiose | C_18_H_32_O_16_ | 5.72 | 0.000 | 5.43 | 2.44 |
| 179.055 | 0.784 | D-Galactose | C_6_H_12_O_6_ | 5.46 | 0.000 | 3.21 | 1.68 |
| 225.061 | 0.784 | β-D-Glucose | C_6_H_12_O_6_ | 5.19 | 0.001 | 3.01 | 1.59 |
| 179.055 | 0.804 | 3-Keto-b-D-galactose | C_6_H_10_O_6_ | 5.17 | 0.000 | 40.47 | 5.34 |
| 1025.340 | 0.784 | Isolactose | C_36_H_66_O_33_ | 5.15 | 0.001 | 0.58 | -0.79 |
| 705.184 | 0.766 | Maltotetraose | C_24_H_42_O_21_ | 4.98 | 0.002 | 1.59 | 0.67 |
| 388.181 | 0.748 | Microcrystalline Cellulose | C_14_H_26_O_11_ | 4.63 | 0.019 | 0.57 | -0.82 |
| 215.016 | 1.054 | Diketogulonic Acid | C_6_H_8_O_7_ | 3.76 | 0.010 | 1.52 | 0.61 |
| 252.108 | 0.694 | Cell Wall Acid | C_9_H_17_NO_7_ | 3.49 | 0.003 | 1.65 | 0.72 |
| 371.083 | 0.784 | 3-O-α-D-Pyranoglucosyl-D-Xylose | C_11_H_18_O_11_ | 3.10 | 0.000 | 4.93×10^14^ | 48.81 |
| 845.277 | 0.764 | 3-O-α-L-Arabinopyranosyl-L-Arabinose | C_30_H_54_O_27_ | 2.90 | 0.000 | 16.86 | 4.08 |
| 204.087 | 0.712 | N-Acetyl-D-glucosamine | C_8_H_15_NO_6_ | 2.78 | 0.006 | 1.42 | 0.50 |
| 213.016 | 0.784 | 5-Phosphoribosyl deoxyribose | C_5_H_11_O_7_P | 2.65 | 0.000 | 3.63×10^14^ | 48.37 |
| 687.174 | 0.748 | A | C_24_H_40_O_20_ | 2.56 | 0.031 | 1.31 | 0.39 |
| 310.113 | 0.712 | N-Acetyl-α-neuraminic Acid | C_11_H_19_NO_9_ | 2.40 | 0.013 | 0.69 | -0.55 |
| 233.006 | 0.694 | Pectin | C_6_H_10_O_7_ | 2.28 | 0.000 | 98.26 | 6.62 |
| 547.152 | 0.805 | B | C_18_H_30_O_16_ | 1.90 | 0.000 | 3.31 | 1.73 |
| 282.118 | 0.694 | N-(1-Deoxy-D-fructosyl) Serine | C_10_H_19_NO_8_ | 1.89 | 0.003 | 1.67 | 0.74 |
| 851.228 | 1.012 | C | C_19_H_22_O_11_ | 1.83 | 0.001 | 1.75×10^14^ | 47.31 |
| 471.136 | 1.095 | 4-O-Methyl-α-D-glucopyranosyl-(1->2)-β-D-xylopyranosyl-(1->4)-D-xylose | C_17_H_28_O_15_ | 1.75 | 0.001 | 3.32 | 1.73 |
| 395.058 | 0.694 | 4-O-α-D-Glucopyranosyl-D-Galactose | C_12_H_20_O_12_ | 1.70 | 0.000 | 31.57 | 4.98 |
| 255.072 | 0.805 | D-altro-Heptose | C_7_H_14_O_7_ | 1.66 | 0.003 | 168.37 | 7.40 |
| 503.162 | 0.784 | α-L-Fucopyranosyl-(1->2)-β-D-pyranogalactosyl-(1->2)-D-Xylose | C_17_H_30_O_14_ | 1.59 | 0.000 | 2.77 | 1.47 |
| 549.168 | 1.159 | Fructooligosaccharide | C_18_H_32_O_16_ | 1.52 | 0.000 | 6.05 | 2.60 |
| 472.276 | 1.054 | Methylcellulose | C_20_H_38_O_11_ | 1.52 | 0.000 | 0.01 | -6.79 |
| 533.172 | 0.745 | Lactosyl Lactose | C_18_H_32_O_15_ | 1.40 | 0.002 | 0.26 | -1.93 |
| 357.104 | 0.669 | D | C_11_H_20_O_10_ | 1.39 | 0.000 | 5.70 | 2.51 |
| 458.187 | 0.730 | Propylene Glycol Alginate | C_17_H_28_O_13_ | 1.33 | 0.004 | 0.20 | -2.29 |
| 217.032 | 0.766 | 3-Dehydro-L-gulonate | C_6_H_10_O_7_ | 1.32 | 0.000 | 716.20 | 9.48 |
| 311.098 | 1.181 | 6-O-α-D-Pyranoglucosyl-D-Glucose | C_11_H_20_O_10_ | 1.27 | 0.000 | 9.03 | 3.17 |
| 473.143 | 5.001 | Lactosyl Glucuronide | C_18_H_24_N_2_O_10_ | 1.26 | 0.004 | 2.07 | 1.05 |
| 268.103 | 0.694 | Neuraminic Acid | C_9_H_17_NO_8_ | 1.14 | 0.029 | 1.44 | 0.52 |
| 632.204 | 0.824 | 3'-Sialyllactose | C_23_H_39_NO_19_ | 1.13 | 0.049 | 1.49 | 0.57 |
| 209.030 | 0.708 | D-Gluconate | C_6_H_10_O_8_ | 1.13 | 0.003 | 0.45 | -1.14 |
| 311.062 | 0.805 | 3-Hydroxy-3-[(3,4,5,6-tetrahydroxyoxan-2-yl)methoxy]propanoic Acid | C_9_H_14_O_9_ | 1.06 | 0.000 | 5.79×10^13^ | 45.72 |
| 666.245 | 0.766 | E | C_24_H_40_O_20_ | 1.02 | 0.033 | 0.74 | -0.44 |
| 517.141 | 0.805 | F | C_18_H_30_O_17_ | 1.00 | 0.000 | 3.89 | 1.96 |

A: 6-{[6-({4,5-dihydroxy-6-methyl-2-[(3,4,5,6-tetrahydroxyoxan-2-yl)methoxy]oxan-3-yl}oxy)-4,5-dihydroxy-2-methyloxan-3-yl]oxy}-3,4,5-trihydroxyoxane-2-carboxylic acid;

B: 6-({4,5-dihydroxy-6-methyl-2-[(3,4,5,6-tetrahydroxyoxan-2-yl)methoxy]oxan-3-yl}oxy)-3,4,5-trihydroxyoxane-2-carboxylic acid;

C: 6-{[3-(1,2-dihydroxybutyl)-1-oxo-1H-isochromen-7-yl]oxy}-3,4,5-trihydroxyoxane-2-carboxylic acid;

D: 6-({[3,4-dihydroxy-4-(hydroxymethyl)oxolan-2-yl]oxy}methyl)oxane-2,3,4,5-tetrol

**Table S3 TK/TW glucoside differential metabolites**

| **m/z** | **Retention time(min)** | **Metabolites** | **Molecular formula** | **VIP** | ***P* value** | **FC** | **log_2_(FC)** |
| --- | --- | --- | --- | --- | --- | --- | --- |
| 387.114 | 0.784 | Maltulose/4-Fructo-α-glucoside | C_12_H_22_O_11_ | 29.59 | 0.002 | 0.77 | -0.38 |
| 379.082 | 0.708 | A | C_21_H_18_O_8_ | 9.58 | 0.000 | 0.55 | -0.85 |
| 363.069 | 0.748 | Aescin | C_15_H_16_O_9_ | 6.78 | 0.004 | 1.36 | 0.44 |
| 525.122 | 0.730 | 5,7,2',3',4'-Pentahydroxyflavone 3,6-dimethyl ether 7-glucoside | C_23_H_24_O_14_ | 4.93 | 0.019 | 1.35 | 0.43 |
| 403.139 | 5.483 | 7-Hydroxyflavanone β-D-pyranoglucoside | C_21_H_22_O_8_ | 4.18 | 0.001 | 0.52 | -0.94 |
| 275.110 | 1.458 | 2,3-Butanediol glucoside | C_10_H_20_O_7_ | 3.51 | 0.006 | 1.70 | 0.77 |
| 797.218 | 0.969 | Sinapyl alcohol 3-O-[2'-(4''-acetyl-rhamnosyl)-6'-glucosyl] glucoside | C_35_H_42_O_21_ | 3.40 | 0.008 | 0.71 | -0.50 |
| 751.212 | 0.969 | Sinapyl alcohol 7-methyl ether 3-neohesperidoside-4'-glucoside | C_34_H_42_O_20_ | 3.07 | 0.013 | 0.73 | -0.45 |
| 723.195 | 0.766 | Menthol glucuronide | C_36_H_36_O_17_ | 2.98 | 0.001 | 1.24 | 0.32 |
| 539.139 | 0.690 | Dihydrocoumarin glucoside | C_23_H_26_O_12_ | 2.71 | 0.000 | 7.61 | 2.93 |
| 296.098 | 0.694 | (S)-Maloyl α-D-glucosamine | C_10_H_17_NO_9_ | 2.59 | 0.000 | 4.83 | 2.27 |
| 315.181 | 5.179 | D-Linalool 3-glucoside | C_16_H_28_O_6_ | 2.41 | 0.000 | 3.37 | 1.75 |
| 371.168 | 4.059 | Anethole glycoside VIII | C_16_H_28_O_8_ | 2.26 | 0.002 | 1.71 | 0.78 |
| 256.139 | 0.804 | (x)-1,2-Propylene glycol 1-O-β-D-pyranoglucoside | C_9_H_18_O_7_ | 2.24 | 0.000 | 1.56×10^4^ | 13.93 |
| 839.228 | 0.906 | Brazilin 7,4'-di(O-glucosyl glucoside) | C_37_H_46_O_23_ | 2.19 | 0.002 | 0.10 | -3.28 |
| 373.183 | 4.168 | (1R,2R,4S)-Menth-1,2,8-triol 8-glucoside | C_16_H_30_O_8_ | 2.11 | 0.013 | 1.96 | 0.97 |
| 459.224 | 5.357 | B | C_21_H_34_O_8_ | 1.90 | 0.017 | 1.34 | 0.42 |
| 549.195 | 4.312 | Phaseolamin E | C_25_H_34_O_12_ | 1.85 | 0.002 | 1.71 | 0.78 |
| 413.217 | 4.819 | Abscisic alcohol 11-glucoside | C_21_H_32_O_8_ | 1.84 | 0.002 | 1.58 | 0.66 |
| 317.197 | 4.935 | Pyrrolidone carboxylic acid menthyl ester | C_16_H_30_O_6_ | 1.83 | 0.002 | 2.27 | 1.19 |
| 439.137 | 7.112 | 4'-Methyllicorice 7-rhamnoside | C_22_H_24_O_8_ | 1.77 | 0.000 | 5.12 | 2.36 |
| 563.210 | 4.312 | C | C_28_H_34_O_12_ | 1.52 | 0.002 | 1.81 | 0.86 |
| 469.205 | 4.102 | Glucogalacto hydroxylysine | C_18_H_34_N_2_O_13_ | 1.51 | 0.001 | 2.94 | 1.56 |
| 485.240 | 6.795 | (3S,7E,9R)-4,7-Megastigmenediene-3,9-diol 9-[apiosyl-(1->6)-glucoside] | C_24_H_40_O_11_ | 1.45 | 0.000 | 0.27 | -1.90 |
| 329.161 | 4.801 | (1S,2S,4R)-Menth-8-ene-1,2,10-triol 2-glucoside | C_16_H_28_O_8_ | 1.44 | 0.001 | 1.81 | 0.86 |
| 387.144 | 5.462 | β-D-Glucoside 4-[(1E)-2-(3-hydroxy-5-methoxyphenyl)ethenyl]benzene | C_21_H_24_O_8_ | 1.42 | 0.023 | 0.55 | -0.86 |
| 503.250 | 5.179 | [6]-Gingerdiol 5-O-β-D-pyranoglucoside | C_23_H_38_O_9_ | 1.40 | 0.005 | 0.41 | -1.30 |
| 509.236 | 6.289 | Gibberellin A37 glucosyl ester | C_26_H_36_O_10_ | 1.39 | 0.001 | 0.36 | -1.49 |
| 527.247 | 5.136 | Cinnamyl alcohol A 19 glucoside | C_26_H_40_O_12_ | 1.36 | 0.000 | 0.02 | -5.76 |
| 355.173 | 4.732 | 7-Hydroxypinostrobin 8-glucoside | C_16_H_28_O_7_ | 1.36 | 0.002 | 1.77 | 0.82 |
| 429.212 | 4.564 | Glucose kiwifruit glucoside | C_19_H_34_O_9_ | 1.35 | 0.002 | 1.50 | 0.59 |
| 557.309 | 8.533 | Capensifolioside | C_30_H_46_O_8_ | 1.35 | 0.017 | 1.50 | 0.59 |
| 431.192 | 4.287 | Chicory butenolide glucoside C | C_19_H_30_O_8_ | 1.34 | 0.014 | 1.46 | 0.54 |
| 473.203 | 4.711 | Dandelion lactone 1-O-B-D-pyranoglucoside | C_21_H_32_O_9_ | 1.33 | 0.014 | 1.48 | 0.57 |
| 401.157 | 4.903 | 3,4,5-Trihydroxy-6-[1-(4-methoxyphenyl)-3-phenylpropoxy]oxane-2-carboxylic acid | C_22_H_26_O_8_ | 1.32 | 0.002 | 2.31 | 1.21 |
| 347.171 | 4.175 | (1S,2S,4S,5S)-2,4,7-Cupressatriol 4-glucoside | C_16_H_28_O_8_ | 1.31 | 0.000 | 2.77 | 1.47 |
| 413.216 | 4.416 | 7,8-Dihydro-3b,6a-dihydroxyα-irone 9-glucoside | C_19_H_34_O_8_ | 1.30 | 0.004 | 1.64 | 0.71 |
| 387.143 | 6.289 | € -4'-Methylresveratrol 3-glucoside | C_21_H_24_O_8_ | 1.30 | 0.000 | 0.32 | -1.64 |
| 355.173 | 4.395 | 6Z-8-Hydroxylinalool 8-O-glucoside | C_16_H_28_O_7_ | 1.24 | 0.000 | 1.66 | 0.73 |
| 543.242 | 4.797 | 3b,6aDihydroxyα-irone 9-[apiosyl-(1->6)-glucoside] | C_24_H_40_O_12_ | 1.24 | 0.011 | 1.30 | 0.38 |
| 711.251 | 4.801 | D | C_32_H_42_O_15_ | 1.22 | 0.030 | 1.40 | 0.49 |
| 591.314 | 5.331 | Deoxycholic acid 3-glucuronide | C_30_H_48_O_10_ | 1.21 | 0.000 | 13.82 | 3.79 |
| 313.166 | 5.134 | (S) -Menth-1-ene-4,7-diol-4-glucoside | C_16_H_28_O_7_ | 1.20 | 0.003 | 1.78 | 0.83 |
| 393.177 | 3.686 | trans-10-Hydroxylinalool oxide 7-glucoside | C_16_H_28_O_8_ | 1.18 | 0.022 | 1.33 | 0.41 |
| 395.192 | 4.175 | (1S,2R,4R,8S)-Menth-2,8,9-triol 2-glucoside | C_16_H_30_O_8_ | 1.17 | 0.002 | 1.82 | 0.86 |
| 621.126 | 1.247 | Calenduloside 3-(6'-p-coumaroyl glucoside) | C_31_H_28_O_15_ | 1.15 | 0.031 | 1.76 | 0.81 |
| 469.111 | 7.003 | Osmanthuside 7-methyl ether 4'-rhamnoside | C_22_H_22_O_10_ | 1.12 | 0.012 | 0.03 | -5.02 |
| 445.147 | 4.710 | 3–(1,1-Dimethylethyl)scopolamine-7-glucoside | C_21_H_26_O_9_ | 1.12 | 0.000 | 4.22 | 2.08 |
| 353.157 | 4.608 | 8-Epilycodial glucoside | C_16_H_26_O_7_ | 1.12 | 0.002 | 2.36 | 1.24 |
| 557.165 | 5.832 | Epicatechin 5-O-β-D-pyranoglucoside-3-benzoate | C_28_H_28_O_12_ | 1.11 | 0.000 | 7.15 | 2.84 |
| 499.218 | 5.592 | Lygodial glucoside tetraacetate | C_24_H_34_O_11_ | 1.11 | 0.011 | 0.71 | -0.50 |
| 371.168 | 3.454 | (1S,2S,4S)-1,8-Epoxy-p-menth-2,7-diol-2-O-b-D-glucoside | C_16_H_28_O_8_ | 1.10 | 0.004 | 2.25 | 1.17 |
| 391.246 | 9.659 | γ-Eudesmol rhamnoside | C_21_H_36_O_5_ | 1.10 | 0.000 | 3.48 | 1.80 |
| 393.177 | 4.041 | Pseudohypericin glycoside | C_16_H_28_O_8_ | 1.09 | 0.023 | 1.36 | 0.44 |
| 411.200 | 4.458 | Fern glycoside D | C_21_H_30_O_8_ | 1.07 | 0.004 | 1.44 | 0.53 |
| 278.123 | 0.766 | 3-Furanmethanolglucoside | C_11_H_16_O_7_ | 1.07 | 0.001 | 1.49 | 0.58 |
| 345.156 | 4.375 | Pseudohypericin lactone | C_16_H_26_O_8_ | 1.06 | 0.004 | 1.80 | 0.85 |
| 270.095 | 1.197 | Linamarin | C_10_H_17_NO_6_ | 1.05 | 0.000 | 1.99 | 1.00 |
| 541.135 | 0.690 | E | C_26_H_24_O_10_ | 1.05 | 0.001 | 3.94 | 1.98 |
| 525.231 | 5.201 | Gibberellin A38 glucoside | C_26_H_36_O_11_ | 1.04 | 0.013 | 0.70 | -0.52 |
| 479.250 | 4.645 | 3-O-(α-L-Rhamnosyl-(1->2)-α-L-rhamnosyl)-3-hydroxydecanoic acid | C_22_H_40_O_11_ | 1.03 | 0.015 | 1.47 | 0.55 |
| 377.182 | 4.398 | 2,6-Dimethyl-6-O-β-D-quinovopyranosyl-7-octadecenoic acid | C_16_H_28_O_7_ | 1.02 | 0.009 | 1.51 | 0.59 |
| 276.108 | 2.249 | Veronica glycoside/4-(β-D-pyranoglucosyloxy)-2-(hydroxymethyl)-2-butylnitrile | C_11_H_17_NO_7_ | 1.02 | 0.000 | 24.99 | 4.64 |
| 499.219 | 5.593 | 6S,9R-Dihydroxy-4,7E-megastigmadien-3-one 9-[apiosyl-(1->6)-glucoside] | C_24_H_38_O_12_ | 1.00 | 0.005 | 0.71 | -0.49 |

A: 6-(3,4-dihydroxy-6-methyl-5-oxooxan-2-yl)-5,7-dihydroxy-2-phenyl-4H-chromen-4-one;

B: (4R,5S,7R,11S)-11,12-Dihydroxy-1(10)-spirovetiven-2-one 11-glucoside;

C: 5,7-Dihydroxy-3',4'-dimethoxy-8-(3-hydroxy-3-methylbutyl)-isoflavone 7-glucoside;

D: 3,4,7-Trihydroxy-5-methoxy-8-prenylflavan 4-O-(beta-D-xylopyranosyl-(1->6)-beta-D-glucopyranoside);

E: 6-({8,8-dimethyl-2-oxo-4-phenyl-2H,8H-pyrano[2,3-f]chromen-5-yl}oxy)-3,4,5-trihydroxyoxane-2-carboxylic acid

**Table S4 TK/TW differential metabolites of organic acids**

| **m/z** | **Retention time(min)** | **Metabolites** | **Molecular formula** | **VIP** | ***P* value** | **FC** | **log_2_(FC)** |
| --- | --- | --- | --- | --- | --- | --- | --- |
| 191.019 | 0.844 | Isocitric Acid | C_6_H_8_O_7_ | 10.05 | 0.004 | 1.73 | 0.79 |
| 191.019 | 1.055 | Citric Acid | C_6_H_8_O_7_ | 7.24 | 0.003 | 1.41 | 0.49 |
| 177.040 | 0.805 | Galacturonic Acid | C_6_H_12_O_7_ | 5.08 | 0.000 | 8.32 | 3.06 |
| 133.050 | 0.804 | Glutaric Acid | C_5_H_8_O_4_ | 2.95 | 0.001 | 9.95 | 3.31 |
| 263.164 | 7.310 | Precocene Acid | C_16_H_24_O_4_ | 2.46 | 0.001 | 0.34 | -1.54 |
| 189.003 | 0.784 | Oxalosuccinic Acid | C_6_H_6_O_7_ | 1.50 | 0.000 | 3.45 | 1.79 |
| 145.013 | 0.824 | Ketoglutaric Acid | C_5_H_6_O_5_ | 1.46 | 0.001 | 21.20 | 4.41 |
| 183.099 | 3.797 | 7-Hydroxyoctanoic Acid | C_8_H_16_O_3_ | 1.40 | 0.001 | 1.94 | 0.96 |
| 205.035 | 0.784 | 3-Oxoadipic Acid | C_6_H_8_O_5_ | 1.29 | 0.000 | 3.32 | 1.73 |
| 113.060 | 1.393 | 3-Methyl-2-oxovaleric Acid | C_6_H_10_O_3_ | 1.14 | 0.011 | 4.13 | 2.05 |
| 227.129 | 4.734 | 3-Hydroxydodecanedioic Acid | C_12_H_22_O_5_ | 1.06 | 0.000 | 2.60 | 1.38 |

**Table S5 TK/TW polyphenol differential metabolites (excluding flavonoids)**

| **m/z** | **Retention time(min)** | **Metabolites** | **Molecular formula** | **VIP** | ***P* value** | **FC** | **log_2_(FC)** |
| --- | --- | --- | --- | --- | --- | --- | --- |
| 119.0858166 | 8.797 | 3-Ethyl-5-methylphenol | C9H12O | 2.13 | 2.44 | 0.42 | 0.05 |
| 123.0806946 | 8.446 | 4-Ethylphenol | C8H10O | 2.07 | 1.25 | -3.38 | 0.00 |
| 119.0858063 | 6.874 | 4-Ethyl-2-methylphenol | C9H12O | 2.05 | 2.61 | 0.59 | 0.01 |
| 373.1285911 | 5.373 | 6-(2,4-Dihydroxyphenyl)-4-methyl-2-(2,3,6-trihydroxyphenyl)cyclohex-3-ene-1-carboxylic acid | C20H20O7 | 1.11 | 1.15 | -1.25 | 0.01 |
| 229.1548362 | 3.776 | Meta-hydroxyisoprenaline | C11H17NO3 | 0.79 | 1.62 | -3.39 | 0.01 |
| 266.1752788 | 5.373 | 4-Hydroxy-allylalcohol | C15H23NO3 | 0.79 | 2.54 | 5.03 | 0.02 |
| 195.1379981 | 6.874 | Hexylresorcinol | C12H18O2 | 0.78 | 2.13 | 1.22 | 0.00 |
| 316.188478 | 5.483 | Vanillyl nonanoate | C17H27NO3 | 0.56 | 1.29 | 1.42 | 0.00 |
| 365.1970998 | 9.565 | 8-Gingerol | C19H28O4 | 0.43 | 1.04 | -2.74 | 0.05 |
| 153.0546422 | 4.840 | Vanillin | C8H8O3 | 0.14 | 2.19 | -2.43 | 0.00 |

**Table S6 TK/TW flavonoid differential metabolites**

| **m/z** | **Retention time(min)** | **Metabolites** | **Molecular formula** | **VIP** | ***P* value** | **FC** | **log_2_(FC)** |
| --- | --- | --- | --- | --- | --- | --- | --- |
| 379.082 | 0.708 | A | C_21_H_18_O_8_ | 9.58 | 0.000 | 0.55 | -0.85 |
| 401.088 | 7.151 | Isoxanthohumol | C_19_H_16_O_7_ | 2.80 | 0.008 | 0.08 | -3.61 |
| 427.066 | 0.764 | (+)-12a-Hydroxyderrisone | C_20_H_14_O_8_ | 2.14 | 0.000 | 0.23 | -2.09 |
| 525.166 | 0.863 | Artelastochromene | C_30_H_30_O_6_ | 2.05 | 0.000 | 2.06 | 1.04 |
| 591.077 | 1.510 | Epigallocatechin 5,7-di-O-gallate | C_29_H_22_O_15_ | 1.86 | 0.012 | 0.86 | -0.22 |
| 399.142 | 4.880 | 2'-Hydroxy-3',4',5',7,8-pentamethoxyflavone | C_20_H_24_O_7_ | 1.72 | 0.000 | 2.25 | 1.17 |
| 443.132 | 5.483 | Villol | C_23_H_22_O_9_ | 1.56 | 0.000 | 0.50 | -1.01 |
| 571.092 | 0.844 | Osmanthuside | C_15_H_10_O_6_ | 1.40 | 0.025 | 1.54 | 0.63 |
| 623.231 | 4.586 | Eruberin B | C_30_H_40_O_15_ | 1.08 | 0.005 | 3.08 | 1.63 |
| 352.082 | 1.262 | Dehydroderrisone | C_19_H_10_O_6_ | 1.07 | 0.027 | 63.67 | 5.99 |

A: 6-(3,4-dihydroxy-6-methyl-5-oxooxan-2-yl)-5,7-dihydroxy-2-phenyl-4H-chromen-4-one

**Table S7 TK/TW alkaloid differential metabolites**

| **m/z** | **Retention time(min)** | **Metabolites** | **Molecular formula** | **VIP** | ***P* value** | **FC** | **log_2_(FC)** |
| --- | --- | --- | --- | --- | --- | --- | --- |
| 729.230 | 0.784 | Citbismine C | C_37_H_36_N_2_O_11_ | 11.52 | 0.001 | 0.72 | -0.46 |
| 373.099 | 0.784 | 7-Hydroxy-6-methyl-8-ribityl lumazine | C_12_H_16_N_4_O_7_ | 3.34 | 0.000 | 171.74 | 7.42 |
| 699.219 | 0.708 | Citbismine F | C_36_H_34_N_2_O_10_ | 2.42 | 0.000 | 70.23 | 6.13 |
| 136.062 | 0.927 | Adenine | C_5_H_5_N_5_ | 2.28 | 0.000 | 0.02 | -5.33 |
| 367.150 | 3.627 | A | C_17_H_22_N_2_O_7_ | 1.70 | 0.004 | 1.34 | 0.42 |
| 424.197 | 5.030 | Neocnidilide | C_21_H_29_NO_8_ | 1.68 | 0.002 | 2.31 | 1.21 |
| 316.189 | 7.310 | Olorofim | C_15_H_18_N_6_O | 1.53 | 0.000 | 11.04 | 3.47 |
| 136.062 | 1.155 | FAPy-adenine | C_5_H_7_N_5_O | 1.52 | 0.000 | 0.04 | -4.61 |
| 137.046 | 1.054 | Hypoxanthine | C_5_H_4_N_4_O | 1.47 | 0.000 | 0.03 | -4.94 |
| 293.136 | 5.052 | Etheno-adenosine | C_12_H_13_N_5_O_3_ | 1.41 | 0.002 | 1.59 | 0.67 |
| 114.092 | 3.907 | 2-Acetylpyrrolidine | C_6_H_11_NO | 1.25 | 0.025 | 0.32 | -1.62 |
| 557.170 | 1.312 | Rutaecarpine | C_17_H_13_NO_3_ | 1.11 | 0.023 | 407.54 | 8.67 |

A :1-(1,2,3,4,5-Pentahydroxypent-1-yl)-1,2,3,4-tetrahydro-beta-carboline-3-carboxylate

| m/z | Retention time(min) | Metabolites | Molecular formula | VIP | *P* value | FC | log_2_(FC) |
| --- | --- | --- | --- | --- | --- | --- | --- |
| 235.169 | 7.003 | Acorusol | C_15_H_24_O_3_ | 24.20 | 0.000 | 3.22 | 1.69 |
| 275.162 | 7.310 | α-Guaiene alcohol | C_15_H_24_O_3_ | 15.24 | 0.000 | 3.46 | 1.79 |
| 235.169 | 7.681 | Zedoary ketone diol | C_15_H_24_O_3_ | 11.64 | 0.000 | 1.82 | 0.86 |
| 235.169 | 4.861 | 2,3-Dihydroabscisic alcohol | C_15_H_24_O_3_ | 11.24 | 0.000 | 2.30 | 1.20 |
| 217.159 | 7.003 | 7-Hydroxy costol | C_15_H_22_O_2_ | 10.77 | 0.000 | 2.03 | 1.02 |
| 273.146 | 6.745 | Abscisic alcohol | C_15_H_22_O_3_ | 9.33 | 0.000 | 1.31 | 0.39 |
| 217.159 | 6.201 | 5beta-1,3,7(11)-Eudesmatrien-8-one | C_15_H_20_O | 8.00 | 0.002 | 1.27 | 0.34 |
| 233.154 | 5.700 | Zingiberene also contains Furanodiene | C_15_H_20_O_2_ | 6.88 | 0.003 | 1.36 | 0.45 |
| 217.159 | 5.483 | Zedoary furanodiene | C_15_H_20_O | 5.58 | 0.004 | 1.34 | 0.42 |
| 189.164 | 7.310 | 1-(2,6,6-Trimethyl-2-cyclohexen-1-yl)-1-penten-3-one | C_14_H_22_O | 5.07 | 0.000 | 3.07 | 1.62 |
| 219.174 | 9.188 | Tanavulgarol | C_15_H_24_O_2_ | 5.00 | 0.011 | 0.77 | -0.37 |
| 305.136 | 5.483 | Claviceps purpurea triterpenol | C_15_H_22_O_5_ | 4.99 | 0.000 | 2.03 | 1.02 |
| 217.159 | 8.797 | Curcumin diacetate | C_15_H_22_O_2_ | 4.46 | 0.008 | 1.35 | 0.44 |
| 251.164 | 5.223 | Ketosantalol | C_15_H_22_O_3_ | 4.31 | 0.000 | 1.68 | 0.75 |
| 203.180 | 9.340 | Rottlerinol II | C_15_H_24_O | 3.87 | 0.000 | 2.96 | 1.57 |
| 219.174 | 6.874 | Hernandulcin | C_15_H_24_O_2_ | 3.43 | 0.000 | 1.64 | 0.72 |
| 219.174 | 5.613 | Aubergenone | C_15_H_24_O_2_ | 3.33 | 0.002 | 1.35 | 0.43 |
| 269.175 | 5.679 | Sweet myrrh curcumol epoxide | C_15_H_24_O_4_ | 3.11 | 0.000 | 2.56 | 1.35 |
| 253.180 | 5.613 | Epoxyrubimycin | C_15_H_24_O_3_ | 3.07 | 0.000 | 2.57 | 1.36 |
| 221.190 | 8.229 | β-Bisabolene alcohol | C_15_H_24_O | 2.97 | 0.000 | 3.47 | 1.79 |
| 219.175 | 10.790 | 3-Hydroxy-1,10-bisaboladien-9-one | C_15_H_24_O_2_ | 2.91 | 0.000 | 3.03 | 1.60 |
| 219.175 | 11.010 | Zedoary alcohol | C_15_H_24_O_2_ | 2.88 | 0.003 | 1.63 | 0.70 |
| 203.180 | 6.811 | Isoeugenol alcohol | C_15_H_24_O | 2.74 | 0.000 | 2.00 | 1.00 |
| 295.188 | 5.483 | 7(14)-Elemadien-2,3,10,11-tetraol | C_15_H_28_O_4_ | 2.40 | 0.000 | 4.57 | 2.19 |
| 233.154 | 6.439 | Zedoary diol | C_15_H_22_O_3_ | 2.21 | 0.019 | 0.84 | -0.26 |
| 253.180 | 6.267 | 6α-Guaiene alcohol | C_15_H_24_O_3_ | 2.13 | 0.000 | 1.89 | 0.92 |
| 305.136 | 5.942 | Pisatin | C_15_H_22_O_5_ | 1.98 | 0.001 | 0.66 | -0.60 |
| 221.190 | 9.703 | Rottlerinol I | C_15_H_24_O | 1.97 | 0.000 | 3.92 | 1.97 |
| 203.180 | 8.510 | Pre-isoshyobunone diol | C_15_H_24_O | 1.93 | 0.001 | 1.92 | 0.94 |
| 201.164 | 8.905 | 4,7(11)-Guaiadien-8-one | C_15_H_22_O | 1.85 | 0.004 | 5.24 | 2.39 |
| 219.175 | 10.415 | Tuberonone | C_15_H_24_O_2_ | 1.74 | 0.003 | 1.39 | 0.48 |
| 249.150 | 10.090 | Valerian terpenol acid | C_15_H_22_O_3_ | 1.71 | 0.006 | 0.01 | -6.82 |
| 298.238 | 7.003 | Valerenol ethyl ether | C_17_H_28_O_3_ | 1.70 | 0.000 | 1.58 | 0.66 |
| 255.196 | 7.681 | Rubimycinol | C_15_H_26_O_3_ | 1.64 | 0.000 | 3.56 | 1.83 |
| 201.164 | 6.874 | α-Dehydroshyobunone | C_15_H_20_ | 1.60 | 0.001 | 2.16 | 1.11 |
| 193.159 | 5.483 | γ-Irone | C_13_H_20_O | 1.53 | 0.000 | 2.54 | 1.34 |
| 280.227 | 8.885 | Santalyl acetate | C_17_H_26_O_2_ | 1.49 | 0.021 | 0.56 | -0.85 |
| 317.173 | 8.251 | Tuberolactone | C_17_H_26_O_4_ | 1.45 | 0.001 | 1.26 | 0.34 |
| 435.199 | 4.732 | 8-Isobutanoylneosolaniol | C_23_H_32_O_9_ | 1.43 | 0.002 | 1.70 | 0.77 |
| 273.125 | 9.467 | α-Cyperone | C_15_H_22_O_2_ | 1.42 | 0.028 | 0.80 | -0.32 |
| 283.154 | 5.136 | Epoxyphaseic acid | C_15_H_22_O_5_ | 1.38 | 0.001 | 1.63 | 0.71 |
| 203.180 | 8.163 | Cineole alcohol | C_15_H_24_O | 1.28 | 0.021 | 1.34 | 0.43 |
| 281.176 | 8.663 | Acoradienol | C_15_H_24_O_2_ | 1.26 | 0.017 | 0.24 | -2.09 |
| 279.193 | 6.439 | Ipomeatetrahydrofuran | C_15_H_28_O_3_ | 1.24 | 0.000 | 1.95 | 0.97 |
| 177.164 | 8.360 | 7,8-Dehydro-3,4-dihydro-β-irone | C_13_H_22_O | 1.13 | 0.005 | 8.75 | 3.13 |
| 265.145 | 8.899 | 13-Nor-6-eudesmen-8,11-dione | C_14_H_20_O_2_ | 1.10 | 0.003 | 0.27 | -1.90 |
| 375.215 | 10.103 | Stevioside C | C_20_H_32_O_5_ | 1.09 | 0.002 | 0.12 | -3.03 |
| 201.164 | 6.007 | α-Dihydroshyobunone | C_15_H_20_ | 1.05 | 0.008 | 1.27 | 0.35 |

**Table S8 Materials related to turmeric**

| **m/z** | **Retention time(min)** | **Metabolites** | **Molecular formula** | **VIP** | ***P* value** | **FC** | **log_2_(FC)** |
| --- | --- | --- | --- | --- | --- | --- | --- |
| 235.169 | 7.681 | Zedoary ketone diol | C15H24O3 | 11.64 | 0.00 | 1.82 | 0.86 |
| 233.154 | 5.700 | Ginger also contains Furanodiene | C15H20O2 | 6.88 | 0.00 | 1.36 | 0.45 |
| 409.162 | 8.926 | Zerumbone B | C22H26O6 | 6.74 | 0.37 | 65.63 | 6.04 |
| 217.159 | 5.483 | Zedoary furanodiene | C15H20O | 5.58 | 0.00 | 1.34 | 0.42 |
| 217.159 | 8.797 | Dehydrocurcumin | C15H22O2 | 4.46 | 0.01 | 1.35 | 0.44 |
| 321.207 | 9.652 | (±)-8-Shogaol | C19H30O4 | 4.28 | 0.01 | 0.00 | -11.63 |
| 269.175 | 5.679 | Sweet myrrh curcumol epoxide | C15H24O4 | 3.11 | 0.00 | 2.56 | 1.35 |
| 219.175 | 11.010 | Eucophylline | C15H24O2 | 2.88 | 0.00 | 1.63 | 0.70 |
| 233.154 | 6.439 | Protozedoarol | C15H22O3 | 2.21 | 0.02 | 0.84 | -0.26 |
| 503.250 | 5.179 | [6]-Gingerdiol 5-O-β-D-pyranoglucoside | C23H38O9 | 1.40 | 0.00 | 0.41 | -1.30 |
| 365.197 | 9.565 | Gingerone /Gingerol | C19H28O4 | 1.04 | 0.01 | 0.15 | -2.74 |
| 365.197 | 9.565 | 8-Gingerone | C19H28O4 | 1.04 | 0.01 | 0.15 | -2.74 |
| 321.205 | 8.273 | 6-Gingerdiol 3-acetate | C19H30O5 | 0.86 | 0.01 | 1.47 | 0.56 |
| 457.245 | 8.071 | 6-Gingerdiol 4'-O-β-D-pyranoglucoside | C23H38O9 | 0.53 | 0.21 | 0.00 | -8.77 |
| 715.280 | 5.312 | 6-Gingersulfonic acid | C17H26O6S | 0.53 | 0.00 | 0.01 | -7.27 |
| 345.204 | 7.507 | (S)-[8]-Gingerol | C19H30O4 | 0.42 | 0.00 | 1.92 | 0.94 |
| 403.210 | 8.186 | [6]-Gingerdiol 3,5-diacetate | C21H32O6 | 0.28 | 0.05 | 0.80 | -0.33 |
| 397.163 | 5.942 | Hexahydrocurcumin | C21H26O6 | 0.27 | 0.00 | 1.67 | 0.74 |
| 353.197 | 8.494 | Methyl gingerol | C18H28O4 | 0.26 | 0.06 | 0.68 | -0.56 |
| 357.170 | 5.395 | Gingerenone A | C21H24O5 | 0.25 | 0.04 | 2.04 | 1.03 |
| 383.208 | 8.085 | 6-Gingerdiol 5-acetate | C19H30O5 | 0.24 | 0.03 | 0.32 | -1.66 |
| 317.173 | 9.703 | Gingerol | C17H26O4 | 0.24 | 0.03 | 1.22 | 0.29 |
| 353.195 | 4.458 | [4]-Gingerdiol 3,5-diacetate | C19H28O6 | 0.19 | 0.01 | 2.47 | 1.30 |
| 513.176 | 5.592 | Dodecylcurcumin | C27H30O11 | 0.06 | 0.13 | 2.03 | 1.02 |
| 397.165 | 4.945 | Dimethoxycurcumin | C23H24O6 | 0.01 | 0.27 | 0.32 | -1.62 |

**Table S9 KEGG pathways and metabolites**

| **KEGG Pathway ID** | **Pathway Name** | **Metabolites Number** | **Metabolites Names (Increase↑/Decrease↓)** | **p-value** |
| --- | --- | --- | --- | --- |
| sce00250 | Alanine, Aspartate and Glutamate Metabolism | 5 | Citrate↑, L-Asparagine↓, L-Glutamate↓, N-Acetylaspartylglutamate↑, Oxoglutarate↑ | 0.00016749 |
| sce00053 | Ascorbate and Aldarate Metabolism | 6 | 3-Dehydro-L-gulonate↑, D-Glucuronate↓, Deoxycholate 3-glucoside↑, Diketogulonate↑, L-(+)-Gulonate↑, Oxoglutarate↑ | 0.000349876 |
| sce00020 | Citric Acid Cycle (TCA Cycle) | 4 | Citrate↑, Isocitrate↑, Oxalosuccinate↑, Oxoadipate↑ | 0.000507872 |
| sce00040 | Pentose and Glucuronate Interconversions | 5 | 3-Dehydro-L-gulonate↑, Deoxycholate 3-glucuronide↑, Diketogulonate↑, Oxoglutarate↑ | 0.004297478 |
| sce00030 | Pentose Phosphate Pathway | 4 | β-D-Glucose↑, 5-Phosphoribosyl deoxyribose↑, Glucuronate↑, Gluconolactone↑ | 0.004415552 |
| sce00220 | Arginine Biosynthesis | 3 | L-Arginine↓, L-Glutamate↓, Oxoglutarate↑ | 0.009591132 |
| sce00630 | Glyoxylate and Dicarboxylate Metabolism | 4 | Citrate↑, Isocitrate↑, L-Glutamate↓, Hydroxyadipate↑ | 0.031905487 |
| sce02010 | ABC Transporters | 5 | Adenosine↓, D-Maltose↑, L-Arginine↓, L-Glutamate ester↓, N-Acetyl-D-glucosamine↑ | 0.033903046 |
| sce00230 | Purine Metabolism | 5 | 5-Hydroxy-2-oxo-4-ureido-2,5-dihydro-1H-imidazole-5-carboxylate↑, Adenine↓, Adenylic acid↓, Guanine↓, Hypoxanthine↓ | 0.036705353 |

Note: ↑ means the content increases after fermentation, ↓ means the content decreases after fermentation.
